# Conserved and divergent functions of Pax6 underlie species-specific neurogenic patterns in the developing amniote brain

**DOI:** 10.1242/dev.159764

**Published:** 2018-04-16

**Authors:** Wataru Yamashita, Masanori Takahashi, Takako Kikkawa, Hitoshi Gotoh, Noriko Osumi, Katsuhiko Ono, Tadashi Nomura

**Affiliations:** 1 Developmental Neurobiology, Kyoto Prefectural University of Medicine, INAMORI Memorial Building, 1-5 Shimogamo-hangi cho, Sakyoku, Kyoto, 606-0823, Japan; 2 Division of Biology, Center for Molecular Medicine, Jichi Medical University, 3311-1 Yakushiji, Shimotsuke, Tochigi, 329-0498, Japan; 3 Department of Developmental Neuroscience, United Center for Advanced Research and Translational Medicine (ART), Tohoku University School of Medicine, 2-1 Seiryo-machi, Aoba-ku, Sendai, Miyagi, 980-8575, Japan

**Keywords:** Amniote, Evolution, Neurogenesis, Pallium, Pax6, Chick, Mouse

## Abstract

The evolution of unique organ structures is associated with changes in conserved developmental programs. However, characterizing the functional conservation and variation of homologous transcription factors (TFs) that dictate species-specific cellular dynamics has remained elusive. Here, we dissect shared and divergent functions of Pax6 during amniote brain development. Comparative functional analyses revealed that the neurogenic function of Pax6 is highly conserved in the developing mouse and chick pallium, whereas stage-specific binary functions of Pax6 in neurogenesis are unique to mouse neuronal progenitors, consistent with Pax6-dependent temporal regulation of Notch signaling. Furthermore, we identified that Pax6-dependent enhancer activity of *Dbx1* is extensively conserved between mammals and chick, although *Dbx1* expression in the developing pallium is highly divergent in these species. Our results suggest that spatiotemporal changes in Pax6-dependent regulatory programs contributed to species-specific neurogenic patterns in mammalian and avian lineages, which underlie the morphological divergence of the amniote pallial architectures.

## Abstract (precis)

**Highlighted Article:** Pax6 promotes neuronal differentiation in the developing chick and mouse telencephalon via Notch inhibition, whereas its stage-specific function in RGC maintenance in the VZ is unique to mammalian neocortical progenitors.

## INTRODUCTION

The evolution of animal body structures is accomplished by pronounced changes in conserved developmental programs. Modifications of regulatory gene networks, such as changes in the expression patterns and dosages of transcription factors (TFs), signaling molecules and their downstream targets, contribute to quantitative and qualitative differences in cellular characteristics and dynamics during embryogenesis (Carroll, 2005; Peter and Davidson, 2011). Recent comparative genomics studies have identified significant differences in *cis-* and *trans-*regulatory elements of developmental regulatory genes that are associated with morphological diversity (Heffer et al., 2010; Glassford et al., 2015; Kvon et al., 2016). By contrast, several lines of evidence demonstrated functional flexibilities of conserved TFs during evolution (Wagner, 2007; Schmidt et al., 2010); however, the variable roles of conserved regulatory genes in taxon- or species-specific developmental programs remain unclear.

The basic patterns of embryonic brain organization are highly conserved in extant vertebrates, whereas the morphology and cellular compositions of mature brains exhibit extensive diversities (Striedter, 2005; Sugahara et al., 2016). In particular, the dorsal part of the telencephalon (the pallium) gives rise to species-specific architecture: the mammalian pallium elaborates the neocortex, which is characterized by horizontal expansion of surface area and a six-layered laminar organization (Nieuwenhuys, 1994). These anatomical hallmarks of the neocortex are constructed by the spatial and temporal regulation of neural progenitor proliferation and differentiation in the developing dorsal pallium; regional and chronological expression of core regulatory genes tightly control the amplification of neural progenitors in the ventricular zone (VZ) and subventricular zone (SVZ) and the sequential production of layer-specific neurons (Götz and Huttner, 2005; Kriegstein et al., 2006). By contrast, the pallium of non-mammalian amniotes, such as reptiles and birds, develops into a three-layered dorsal cortex or Wulst with a tissue slab (Ulinski, 1990; Medina and Reiner, 2000). Furthermore, reptiles and birds have a dorsal ventricular ridge, which is a prominent tissue protrusion at the lateral wall of the cerebral hemisphere, as a derivative of the ventral pallium (VP) (Ulinski, 1983). These morphological differences might be provided by species-specific patterns of progenitor proliferation and neuronal specification in distinct sectors of the embryonic pallium (Suzuki et al., 2012; Nomura et al., 2013; García-Moreno and Molnár, 2015).

Pax6 is a paired domain-containing TF that is expressed in region-specific neural progenitor cells in the developing vertebrate central nervous system (Osumi et al., 2008; Manuel et al., 2015; Ypsilanti and Rubenstein, 2016). Consistently, Pax6 regulates various downstream target genes in response to cell-intrinsic and extrinsic signaling, as well as in response to the levels of Pax6 itself in neuronal progenitors (Holm et al., 2007; Sansom et al., 2009; Thakurela et al., 2016). The expression pattern of Pax6 is highly conserved among vertebrates (Fernandez et al., 1998; Puelles et al., 2000). However, recent studies have demonstrated species-specific functions of Pax6 in the regulation of neural progenitors in the developing mammalian brain (Zhang et al., 2010; Wong et al., 2015), suggesting that evolutionarily variable functions of Pax6 underlie the divergent gene regulations and cellular compositions in the developing pallium (Molnár and Butler, 2002; Aboitiz and Zamorano, 2013). Consistently, Pax6 is required for the establishment of the pallial-subpallial boundary by regulating specific target gene expression in the VP (Yun et al., 2001; Assimacopoulos et al., 2003), a crucial region for the taxon-specific pallial architectures in amniotes (Molnár and Butler, 2002).

Here, we report shared and divergent functions of Pax6 in the regulation of species-specific cellular dynamics and downstream target genes in developing mammalian and avian brains. Genetic manipulation of Pax6 functions in the developing chick pallium revealed a conserved role of Pax6 in promoting neuronal differentiation in both mouse and chick. By contrast, Pax6-dependent maintenance of neural progenitors is unique to mammalian corticogenesis, which is consistent with the temporal regulation of Notch signaling activities. Furthermore, we identified that Pax6 has the potential to activate *Dbx1* expression in the developing avian pallium, implicating that gene regulation unique to the mammalian pallium is extensively conserved in non-mammalian brain development. Our results suggest that lineage-specific changes in Pax6-dependent gene regulation contribute to the establishment of the mammalian and avian neurogenic programs, on the basis of conserved regulatory mechanisms derived from common ancestors of amniotes.

## RESULTS

### Genome editing in chick brain reveals conserved functions of Pax6 in neurogenesis

**Fig. 1.**
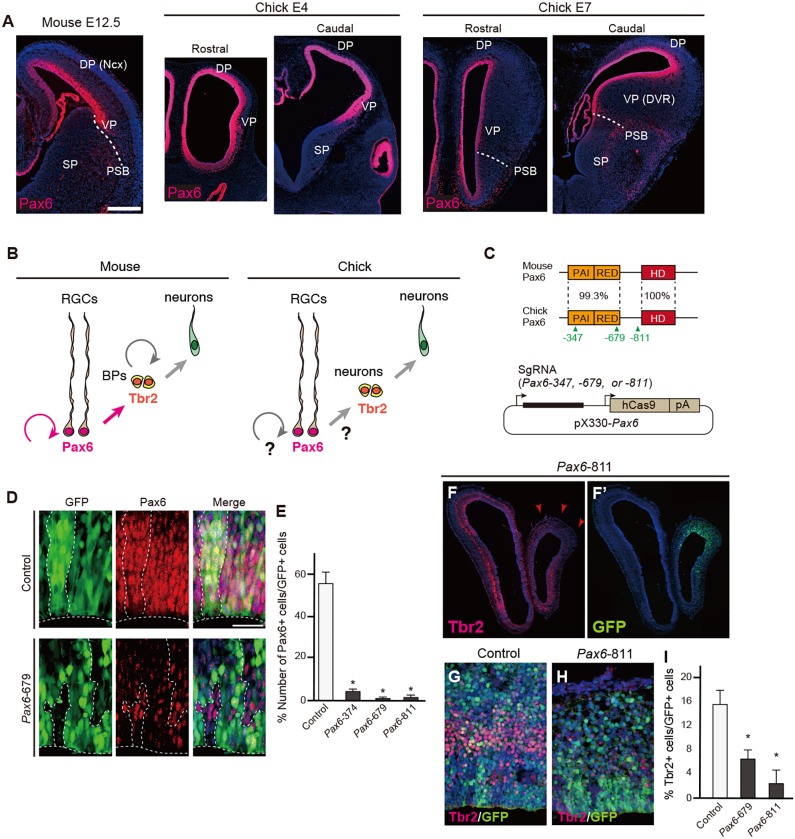
**Targeted deletion of the endogenous *Pax6* gene in the developing chick pallium.** (A) Expression patterns of Pax6 protein in mouse (E12.5) or chick (E4 and E7) telencephalon. DP, dorsal pallium; Ncx, neocortex; PSB, pallium-subpallium boundary; SP, subpallium; VP, ventral pallium; DVR, dorsal ventricular ridge. (B) Interspecies differences in mouse and chick pallial neurogenesis. BPs, basal progenitors; RGCs, radial glial cells. (C) (Top) Protein structures of mouse and chick Pax6 showing the percentage identity of domains. The paired domain consists of PAI and RED subdomains; HD, homeodomain. The location of the three sgRNA target sites is indicated. (Bottom) The *pX330-Pax6* vector for simultaneous expression of sgRNA and Cas9. (D,E) Electroporation of *pX330-*based vectors into the developing chick pallium. The number of Pax6-positive cells among GFP-positive cells is significantly decreased by electroporation with *pX330-Pax6* vectors (E). Welch's *t*-test, *P<0.05. Error bars indicate s.e.m. *n*=4. (F-I) Decreased number of Tbr2-positive cells after electroporation of *pX330*-*Pax6*. Arrowheads indicate the reduction of Tbr2 expression in the electroporated region. There was no significant difference in the proportion of Tbr2-positive cells between *Pax6*-679 and -811, indicating that the two sgRNAs were equally efficient in targeting chick Pax6. Error bars indicate s.e.m. *n*=4 for control, *n*=3 for *Pax6*-679 and -871 samples. Two-tailed Student's *t*-test, *P<0.05. Scale bars: 200 µm in A; 25 µm in D.

We chose Pax6 for the analyses of conserved and derived roles of homologous TFs in species-specific brain development because (1) the protein structures and expression patterns of Pax6 are extremely highly conserved among species, (2) other genes with compensatory functions are not expressed in the developing pallium, and (3) downstream target genes have been well-characterized in the developing mouse neocortex. In the developing mouse and chick pallium, Pax6 is highly expressed in the VZ neural progenitors [radial glial cells (RGCs)], although the progenitor compositions and characteristics are not identical in these species (Fig. 1A,B). Notably, despite conservation of Tbr2 (Eomes) expression, Tbr2-positive cells are not basal progenitors but postmitotic neurons in the developing chick pallium (Nomura et al., 2016).

To dissect conserved and variable functions of Pax6 in species-specific brain development, we first performed *in vivo* targeting of chick Pax6 by CRISPR/Cas9-mediated genome editing. We designed three single-guide RNAs (sgRNAs) against the coding region of the chick *Pax6* gene (*Pax6*-347, *Pax6*-679 and *Pax6*-811) and cloned them into *pX330* vectors that simultaneously express Cas9 and each sgRNA under the control of different promoters (Fig. 1C, Fig. S1A). These *Pax6* targeting vectors, together with a GFP reporter vector, were electroporated into the pallium of developing chick embryos at embryonic day (E) 4 (which corresponds to Hamburger Hamilton stage 23-24). At 36 h after electroporation, significant decreases in Pax6 expression levels were evident in the neural progenitors transfected with *pX330*-*Pax6*-347, -679 or -811 compared with those transfected with control plasmid (*pX330* without sgRNA) (Fig. 1D,E, Fig. S1C). High-throughput genome sequencing also confirmed successful insertion-deletion mutations at the target sequences of Pax6 in GFP-positive cells (Fig. S1B).

At 48 h after electroporation, we confirmed that the number of Tbr2-positive or Tbr1-positive postmitotic neurons was dramatically decreased in embryos transfected with *pX330*-*Pax6*-679 or -811 vectors compared with control embryos (*n*=3 embryos; Fig. 1F-I, Fig. S1F,F′). Electroporation of *pX330*-*Pax6*-679 or -811 also induced ectopic expression of Gsh2, a marker for the embryonic subpallium, in the lateral and ventral pallium (Fig. S1D-E′; *n*=3 of 4 embryos). At later embryonic stages, ectopic accumulation of gamma-aminobutyric acid (Gaba)-positive cells was evident at the VP transfected with *pX330*-679, suggesting that ectopic Gsh2-positive cells differentiate into inhibitory interneurons (Fig. S1J,K). These phenotypes resembled the pallium of *Pax6* mutant mice and rats (Toresson et al., 2000; Yun et al., 2001; Kroll and O'Leary, 2005; Nomura et al., 2006) (Fig. S1H,I). Electroporation of *pX330*-*Pax6*-679 did not increase the number of active caspase 3-positive apoptotic cells (Fig. S1L-N). Thus, Pax6 has a highly conserved function in the differentiation of excitatory neurons from pallial RGCs, despite the divergence of pallial progenitor compositions.

### Stage-dependent functions of Pax6 are unique to mammalian neural progenitors

**Fig. 2.**
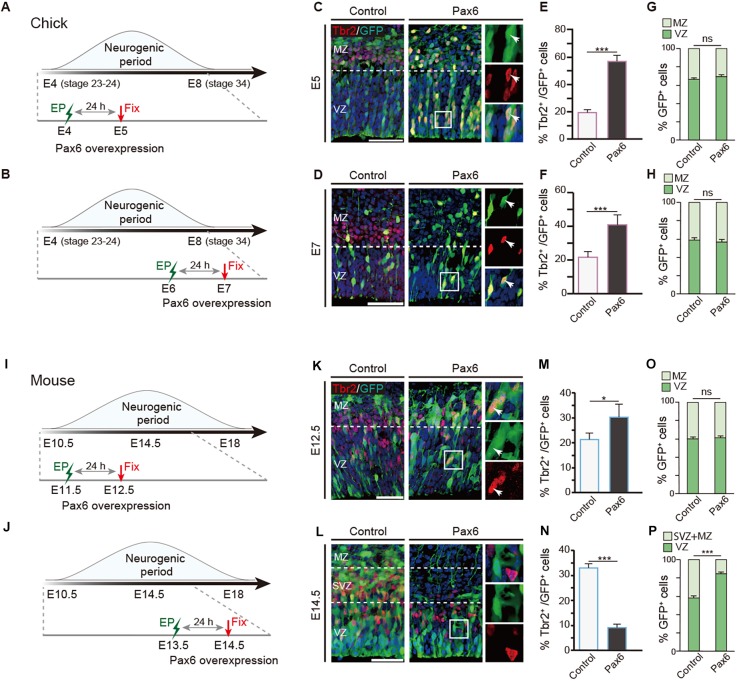
**Interspecies differences in Pax6-dependent pallial neurogenesis between mouse and chick.** (A,B) Time schedules of electroporation (EP) in the developing chick pallium. (C-H) Distributions of GFP-positive cells and Tbr2-positive cells in the developing mouse and chick pallium (C,E,G, E5; D,F,H, E7) electroporated with control and Pax6 expression vectors. Pax6 overexpression increases Tbr2-positive cells in both the E5 and E7 chick pallium. Boxed regions are shown at higher magnification on the right, in single channel and merge. Arrowheads indicate cells double positive for Tbr2 and GFP. (I,J) Time schedules of electroporation in the developing mouse pallium. (K-P) Distributions of GFP-positive cells and Tbr2-positive cells in the developing mouse neocortex (K,M,O, E12.5; L,N,P, E14.5) electroporated with control and Pax6 expression vectors. Error bars indicate s.e.m. *n*=6 for each case. Two-tailed Student's *t*-test, *P<0.05, ***P<0.005; ns, not significant. VZ, ventricular zone; SVZ, subventricular zone; MZ, mantle zone. Scale bars: 50 µm.

Pax6 plays opposing roles in neurogenesis, either promoting or inhibiting neuronal differentiation in the developing mouse neocortex in stage- and dose-dependent manners (Fukuda et al., 2000; Estivilli-Torrus et al., 2002; Sansom et al., 2009; Tuoc et al., 2009). To address whether these binary functions of Pax6 are evolutionarily conserved among amniotes, we performed gain-of-function analyses of Pax6 in chick pallial neural progenitors at different embryonic stages. Expression vectors for *Pax6* and *GFP* were electroporated into the developing chick dorsal pallium at E4 (stage 23-24) or E6 (stage 28-29), which correspond to the early and middle/late stages of neurogenesis, respectively (Fig. 2A,B). At 24 h after electroporation, Pax6 overexpression dramatically increased the proportion of Tbr2-positive cells compared with control embryos transfected with the GFP expression vector, at both early and late stages of neurogenesis (Fig. 2C-F). The Tbr2-positive cells in the chick pallium did not show any proliferative activity, indicating that they are postmitotic neurons, as previously reported (Nomura et al., 2016) (data not shown). Intriguingly, Pax6 overexpression did not alter the proportion of transfected cells in the VZ, suggesting that high-dose Pax6 induced premature neuronal differentiation of progenitors prior to exiting the VZ (Fig. 2G,H).

To examine changes in the frequency of proliferative and neurogenic divisions due to high-dose Pax6, we performed clonal analysis of chick pallial progenitors by co-electroporation of *Cytbow/Nucbow* and Cre recombinase expression vectors, as previously reported (Loulier et al., 2014). At E5, 24 h after electroporation, Pax6 overexpression reduced the proportion of clones containing Sox2-positive progenitors and increased clones with Sox2-negative non-progenitors, suggesting that high-dose Pax6 facilitated symmetric and asymmetric neurogenic divisions (Fig. S2). Thus, consistent with the loss-of-function study, Pax6 plays a crucial role in promoting neuronal differentiation in the developing chick pallium in both the early and late neurogenic periods.

By contrast, in the developing mouse pallium, high levels of Pax6 expression induced distinct outcomes in neuronal progenitors depending on the embryonic stage. In E12.5 mice, overexpression of Pax6 significantly increased the number of Tbr2-positive cells compared with GFP-electroporated control embryos (Fig. 2I,K,M,O). By contrast, in the E14.5 mouse pallium, high-dose Pax6 decreased the number of Tbr2-positive cells at the VZ and SVZ while significantly increasing the proportion of RGCs at the VZ (Fig. 2J,L,N,P).

To examine the proliferative activity of transfected cells in the VZ, we administered EdU 1 h before fixation. In the E12.5 mouse pallium, overexpression of Pax6 significantly decreased the proportion of EdU-positive neural progenitor cells in the VZ (Fig. S3A). However, at middle/later stages of neurogenesis, high-dose Pax6 did not change the proportion of EdU-positive cells in the VZ (Fig. S3B,C), indicating that the proliferative activity of RGCs was maintained by Pax6 overexpression. Similar alterations in progenitor proliferation were also evident in the developing chick pallium by overexpression of Pax6 or a dominant-negative form of Pax6 with the repressor domain (EnR) of the *engrailed* gene (Fig. S3D-F).

These data demonstrated that Pax6 has a conserved function in progenitor proliferation in both the mouse and chick pallium. By contrast, stage-specific binary functions of Pax6 in neuronal differentiation are unique to mouse neocortical neuronal progenitors: high-dose Pax6 accelerates neuronal commitment or differentiation in early stages, whereas it suppresses neuronal differentiation and maintains RGCs after the middle stage of corticogenesis.

### Interspecies differences in Pax6-dependent regulation of Notch signaling

**Fig. 3.**
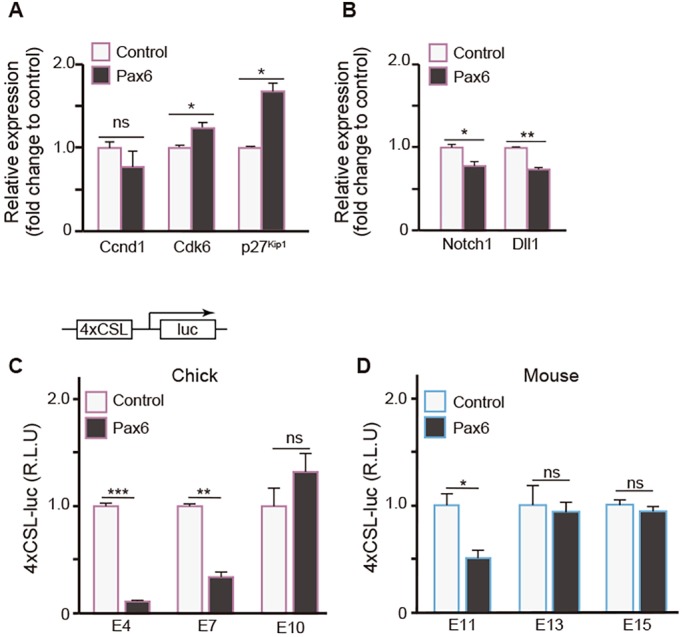
**Temporal differences in Pax6-dependent regulation of Notch signaling.** (A,B) mRNA expression levels of *Ccnd1*, *Cdk6*, *p27^Kip1^*, *Notch1* and *Dll1* in E5 chick pallium electroporated with control or Pax6 expression vectors. *n*=3. (C,D) Pax6-dependent temporal changes in Notch reporter (*p4xCSL*-luciferase) activity in neuronal progenitors from different embryonic stages of chick (C) and mouse (D) dorsal pallium. *n*=4 for each case. R.L.U, relative luciferase units. (A-D) Error bars indicate s.e.m. Two-tailed Student's *t*-test, *P<0.05, **P<0.01, ***P<0.005.

To investigate the regulatory mechanisms underlying conserved and variable functions of Pax6 in neurogenesis, we examined gene expression directed by Pax6 in chick pallial progenitors. Quantitative PCR (qPCR) demonstrated that Pax6 overexpression significantly increased the expression of genes encoding Cdk6 and p27^Kip1^ [also known as cyclin-dependent kinase inhibitor 1B (Cdkn1b)] (Fig. 3A), which are regulators of cell cycle progression as downstream targets of Pax6 (Duparc et al., 2007; Sansom et al., 2009). By contrast, high-dose Pax6 decreased the expression of genes encoding Notch1 receptor and Dll1 ligand in the E5 chick pallium, suggesting that Pax6 overexpression altered Notch signaling activity (Fig. 3B).

To address whether interspecies differences in neurogenic functions of Pax6 are mediated by Notch signaling, we examined Pax6-dependent Notch signaling activity at distinct stages of the developing chick and mouse pallium by transfection of a Notch reporter construct (*4xCSL-luc*) together with the *Pax6* expression vector into isolated neural progenitors (Fig. 3C,D, Fig. S4). After 24 h of transfection, we confirmed that Pax6 overexpression significantly decreased Notch reporter activity in both the early (E4) and late (E7) stages of chick neural progenitors (Fig. 3C). By contrast, Pax6-dependent downregulation of Notch reporter activity was only detected at the early stage (E11) but not at the middle (E13) and late (E15) stages of mouse pallial neural progenitors (Fig. 3D). This is in agreement with the stage-specific changes in neural progenitor states induced by Pax6 overexpression in the developing mouse pallium. Notably, a decrease in Notch activity due to Pax6 was not evident in progenitors isolated from the E10 chick pallium (Fig. 3C), when neurogenesis in the developing chick pallium is almost complete (Tsai et al., 1981). This suggests that Pax6-dependent temporal changes in Notch signaling are shared between mouse and chick pallial progenitors, which are uncoupled from the neurogenic periods in each species.

### Neural progenitors in the chick VP are less sensitive to high-dose Pax6

Recent studies have shown that spatial differences in pallial neurogenic potentials underlie species-specific neuron subtype compositions (Suzuki et al., 2012; García-Moreno et al., 2018). To examine whether neural progenitors in distinct pallial regions exhibit differential responses to Pax6, we overexpressed Pax6 in the VP of the developing chick brain. At E5, 24 h after electroporation, high-dose Pax6 reduced the proportion of EdU-positive cells in the VP (Fig. S5). By contrast, there was no difference in the number of Tbr2-positive cells in the VP transfected with control and Pax6 expression vectors, suggesting that high-dose Pax6 does not promote neural differentiation in the VP. We also examined Pax6-dependent Notch activity in neurospheres isolated from the chick VP. Pax6 overexpression decreased Notch reporter activity in both the early (E4) and late (E7) stages of VP-derived neural progenitors, but to a lesser degree than in the dorsal pallium-derived neurospheres (Fig. S5).

These results demonstrate regional differences in neural progenitors of the developing chick pallium; neural progenitors derived from the VP are less sensitive to high-dose Pax6 than those from the dorsal pallium with respect to neurogenic potentials. Notably, reduced Notch activity due to Pax6 was not detected in progenitors derived from the E10 VP (Fig. S5), as was also the case for the dorsal pallium, indicating that Pax6-dependent temporal changes in Notch activity are common characteristics of the dorsal and ventral pallial progenitors in chick.

### High-dose Pax6 activates *Dbx1* expression in the developing chick pallium

**Fig. 4.**
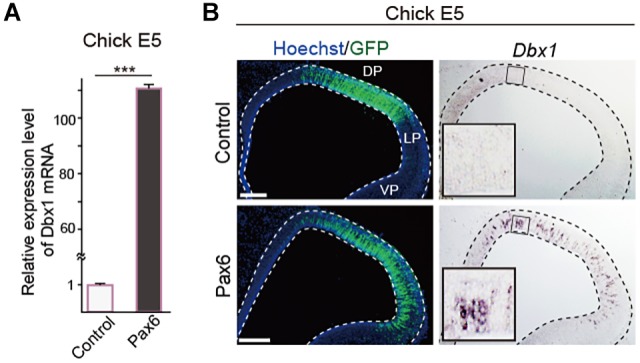
**Increased Pax6 level activates *Dbx1* expression in the developing chick pallium.** (A) qRT-PCR demonstrates a significant increase in *Dbx1* expression in the E5 chick pallium upon overexpression of Pax6. Error bars indicate s.e.m. *n*=3. Two-tailed Student's *t*-test, ***P<0.05. (B) *In situ* hybridization of *Dbx1* mRNA (right) in the E5 chick dorsal (DP) and lateral (LP) pallium electroporated with control or *Pax6* expression vectors. Scale bars: 200 µm.

To further investigate shared and divergent gene regulations directed by Pax6 in an unbiased manner, we performed comprehensive transcriptome analyses for Pax6 target genes in the developing chick pallium. We overexpressed Pax6 in the E5 chick pallium and alterations in mRNA expression profiles were analyzed by RNA sequencing (RNA-seq). As a control, only the GFP reporter vector was introduced into the chick pallium. Pax6 overexpression altered the expression of various genes in the developing chick pallium, including previously reported Pax6 target genes (Table S1). Among these, *Dbx1* exhibited the highest fold change in response to Pax6 overexpression (Table 1). *Dbx1* encodes a homeobox-containing TF that is expressed in the developing mammalian brain and spinal cord under the control of Pax6 (Takahashi and Osumi, 2002; Carney et al., 2009; Numayama-Tsuruta et al., 2010). In the embryonic mouse telencephalon, *Dbx1* is expressed in the septum and VP, and it plays essential roles in the production of specific neuron subtypes including Cajal-Retzius cells and excitatory neurons that migrate into the neocortex or amygdala (Bielle et al., 2005; Hirata et al., 2009; Teissier et al., 2010; Puelles et al., 2016). qPCR analysis confirmed a significant increase in *Dbx1* expression induced by high-dose Pax6 in the developing chick pallium (Fig. 4A). Furthermore, *in situ* hybridization against chick *Dbx1* mRNA demonstrated that Pax6 overexpression induced *Dbx1* expression in the broad area of the chick pallium (Fig. 4B, Fig. S6A,A′). Thus, *Dbx1* is a common downstream target gene of Pax6 in the developing mammalian and avian pallium.

**Table 1.**
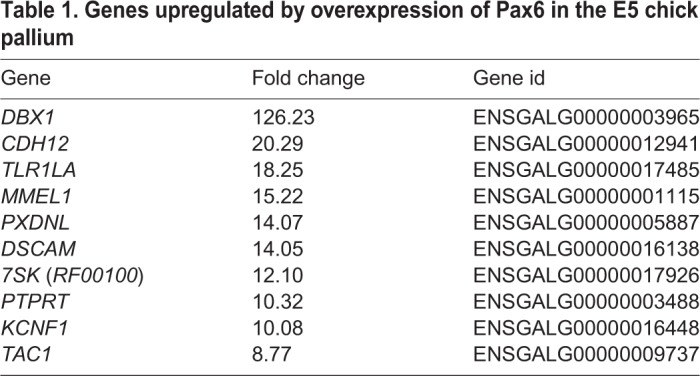
**Genes upregulated by overexpression of Pax6 in the E5 chick pallium**

### Enhancer activity driving *Dbx1* expression in the VP is highly conserved among amniotes

**Fig. 5.**
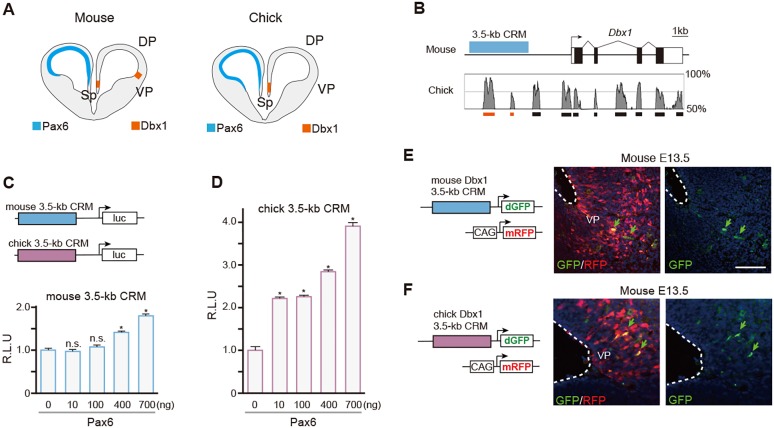
**Evolutionarily conserved enhancer activity of the *Dbx1* 3.5-kb CRM in the developing mouse and chick pallium.** (A) Illustration of *Dbx1* and Pax6 expression in the developing mouse and chick telencephalon. Sp, septum. (B) Comparison of the genomic sequences of the mouse and chick *Dbx1* loci. Conserved sequences (boxes at bottom) within the *Dbx1* 3.5-kb CRMs are indicated in orange. (C,D) Luciferase assays with mouse (C) and chick (D) *Dbx1* 3.5-kb CRMs in HEK293T cells. Error bars indicate s.e.m. *n*=4 for each case. Dunnett's multiple comparison test, *P<0.05. R.L.U, relative luciferase units. (E,F) The expression of destabilized GFP (dGFP) driven by mouse (E) or chick (F) *Dbx1* 3.5-kb CRM in the developing mouse pallium (E13.5). dGFP is specifically expressed in the VP (arrows). Scale bar: 100 µm.

Notably, the expression pattern of *Dbx1* in the developing pallium is not conserved among amniotes: *Dbx1* is expressed at the septum but is absent at the VP in developing avian brain (Bielle et al., 2005; Nomura et al., 2008), whereas Pax6 is highly expressed in the VP (Fig. 5A). To address whether the Pax6-dependent induction of *Dbx1* in the developing chick pallium is mediated by conserved regulatory mechanisms, we performed comparative functional analyses of mouse and chick *Dbx1 cis*-regulatory regions. A previous study reported that the distal 3.5 kb of the *Dbx1* regulatory region acts as a *cis*-regulatory module (CRM) that is sufficient to drive specific *Dbx1* expression in the developing mouse pallium (Lu et al., 1996). Because this regulatory region contains sequences that are highly conserved among amniotes (Fig. 5B, Table S2), we isolated corresponding 3.5 kb fragments from both mouse and chick *Dbx1* genomic loci (termed the *Dbx1* 3.5-kb CRM) and examined their Pax6-dependent transcriptional activities. Co-transfection of the Pax6 expression vector and luciferase expression vector containing mouse or chick *Dbx1* 3.5-kb CRM into HEK293T cells significantly increased luciferase activities in a Pax6 dose-dependent manner (Fig. 5C,D). Notably, the chick *Dbx1* 3.5-kb CRM exhibited more pronounced transcriptional activity than the mouse *Dbx1* 3.5-kb CRM for the same amount of Pax6 (Fig. 5D). Transient chromatin immunoprecipitation (ChIP) assays demonstrated significant enrichment of Pax6 at putative Pax6 binding sites (Pax6 BS1-BS7) in the chick *Dbx1* 3.5-kb CRM in HEK293T cells (Fig. S6B,C). Thus, both chick and mouse *Dbx1* 3.5-kb CRMs have conserved transcriptional activities in response to Pax6.

These lines of evidence suggest that the enhancer activity of the CRM that drives *Dbx1* expression in the developing VP is also highly conserved between mouse and chick. To test this, we generated reporter constructs expressing destabilized GFP (dGFP) under the control of either mouse or chick *Dbx1* 3.5-kb CRM, and electroporated these constructs into the developing mouse telencephalon. *pCAG-mRFP* was co-transfected to monitor transfection efficiency. At 24 h after electroporation, we confirmed the induction of dGFP expression by mouse *Dbx1* 3.5-kb CRM in the developing mouse VP, which faithfully recapitulated endogenous *Dbx1* expression in the developing mouse pallium (*n*=4, Fig. 5E). Surprisingly, introduction of the reporter vector with chick *Dbx1* 3.5-kb CRM also induced dGFP expression in the mouse VP (*n*=3, Fig. 5F). Thus, the enhancer activity that drives *Dbx1* expression in the mammalian VP is extensively conserved among amniotes, even though endogenous *Dbx1* expression is not detected in the developing non-mammalian VP.

## DISCUSSION

**Fig. 6.**
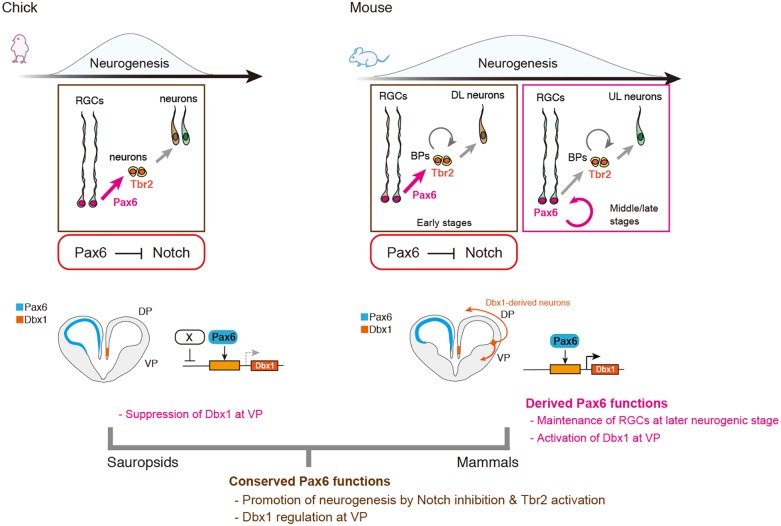
**Conserved and derived functions of Pax6 underlying species-specific neurogenic programs in the developing mouse and chick pallium.** (Top) Pax6-dependent suppression of Notch signaling and promotion of neuronal differentiation are highly conserved in developing chick and mouse dorsal pallial progenitors. Conversely, Pax6-dependent maintenance of RGCs at middle/late neurogenic stages is unique to the mouse dorsal pallium (neocortex), which might be a derived Pax6 function in the mammalian lineage. It has also been suggested that fate-restricted RGCs (in different colors) contribute to distinct neuronal subtypes, such as deep (DL) or upper (UL) layer neurons, although our study did not distinguish the heterogeneity of RGCs. Notably, chick ventral pallial progenitors are less sensitive to high doses of Pax6. (Bottom) Structural and functional conservations of *Dbx1* 3.5-kb CRMs suggest that the regulatory mechanism for *Dbx1* expression in the mammalian VP had evolved before the split of mammalian and non-mammalian (sauropsid) lineages. Dbx1 overexpression is not sufficient to generate tangentially migrating glutamatergic neurons in the developing chick VP (Garcia-Moreno et al., 2018). Thus, in mammals, activation of Dbx1 expression, as well as additional regulatory networks in the VP, underlie the generation of unique neuronal populations essential for producing mammalian-type pallial structures.

It has been proposed that variable functions of Pax6 and its downstream effectors are crucial in the evolution of the mammalian neocortex (Molnár and Butler, 2002; Aboitiz, 2011; Aboitiz and Zamorano, 2013), although functional conservation and variation of Pax6 have not been empirically addressed. Here, we clarified that Pax6 plays common roles in promoting neuronal differentiation in the developing chick and mouse telencephalon, whereas stage-specific binary functions of Pax6 are not conserved; in particular, Pax6-dependent maintenance of RGCs in the VZ is unique to mammalian neocortical progenitors at the middle and late embryonic stages (Fig. 6). Interspecies differences in stage-specific neurogenic functions of Pax6 are correlated with temporal regulation of Notch signaling activity. Intriguingly, E11 mouse cortical neural progenitors and E4 and E7 chick pallial neural progenitors exhibited similar responses to functional manipulation of Pax6, including Pax6-dependent negative regulation of Notch signaling. By contrast, Pax6-dependent suppression of Notch signaling was attenuated in E13 and E15 mouse cortical progenitors and E10 chick dorsal pallial progenitors; the former generate later-born cortical neurons, whereas the latter contribute to gliogenesis (Tsai et al., 1981; Cheung et al., 2007). Thus, the mammalian-specific function of Pax6 in the maintenance of RGCs at the later stages of cortical development might be a consequence of the relative shift of the neurogenic period to Pax6-dependent regulatory programs. Time-dependent regulations of the progenitor state by Pax6 are evolutionarily conserved among amniotes, while extension of the neurogenic period provides a mammalian-specific function of Pax6 at the middle and late embryonic stages (Fig. 6). Temporal changes in the Pax6-dependent neurogenic properties of mammalian neural progenitors are linked with the time-dependent regulation of laminar-specific neuron production in the developing mammalian neocortex. Accordingly, the progenitor potential in middle and late corticogenesis is restricted to generating upper cortical neurons that constitute interhemispheric connections, which are thought to be an evolutionary novelty in mammals (Suárez et al., 2014). Thus, it is possible that the Pax6 function in later corticogenesis that maintains progenitor pools was recruited to reserve mammalian-specific fate-committed progenitors during the evolution of mammalian lineages. Consistently, sustained Pax6 expression induced massive production of upper layer cortical neurons derived from Pax6-positive RGCs in the outer SVZ, which underlies the evolutionary expansion of the primate neocortex (Wong et al., 2015).

Several studies have also shown spatiotemporal differences in the neurogenic potential of RGCs in the developing chick pallium (Striedter and Beydler, 1997; Suzuki et al., 2012). Notably, we identified regional differences in the response to high-dose Pax6 between the dorsal and ventral pallium, which are inversely correlated with the medial (dorsal)-low and lateral (ventral)-high neurogenic gradient of the developing chick pallium (Suzuki et al., 2012). Thus, spatially biased neurogenic potentials of chick pallial neural progenitors might constrain the responsiveness to high-dose Pax6. We could not detect obvious differences in the expression level of endogenous Pax6 protein between the dorsal and ventral pallium in chick brain (Fig. 1A), suggesting that additional mechanisms underlie regional differences in progenitor properties.

Interspecies differences in TF binding sites cannot be predicted by genome sequence alignments (Wilson and Odom, 2009). Here, we identified that the chick *Dbx1* 3.5-kb CRM responds to high-dose Pax6 and it retains cryptic enhancer activity to drive reporter gene expression in the developing mouse VP. This provides a novel example in which divergent gene expression is not driven by the simple gain or loss of CRMs. The regulatory mechanisms responsible for the interspecies differences in *Dbx1* expression remain to be elucidated. Induction of *Dbx1* by high-dose Pax6 suggests that the expression level of endogenous Pax6 in the developing chick pallium is below the threshold of *Dbx1* activation; however, we disfavor this possibility because (1) quantitative and qualitative analyses of *Pax6* transcripts revealed comparable levels in the developing mouse and chick pallium (Fig. 1A; data not shown), (2) our *in vitro* luciferase assay indicated greater transcriptional activity of the chick than mouse *Dbx1* 3.5-kb CRM in the presence of the same amount of Pax6, and (3) the *in vivo* reporter assay demonstrated strong transcriptional activities of mouse and chick *Dbx1* 3.5-kb CRMs in the developing chick pallium (Fig. S7A,B). These results suggest that species-specific differences in *Dbx1* expression might be due to additional suppressive mechanisms mediated by sequence differences in other genomic regions or distinct epigenetic signatures (Fig. 6) (García-Moreno et al., 2018). Several studies have shown that progressive changes in the composition of BAF (Brg1/Brm-associated factor) subunits confer regulation of Pax6 target genes in the developing mammalian neocortex (Ninkovic et al., 2013; Tuoc et al., 2013; Bachmann et al., 2016). Functional association of these chromatin-remodeling factors with Pax6 in the developing non-mammalian brain remains to be elucidated.

The extensive conservation of Pax6-dependent *Dbx1* regulation suggests that this regulatory mechanism derived from a common ancestor of amniotes (Fig. 6). Although the anatomical structure of ancestral amniote brains remains unknown, it is noteworthy that a part of the genetic program specific for neocortical development is an ancestral character of amniotes. Pax6-dependent *Dbx1* expression in the developing spinal cord is highly conserved among vertebrates, and we confirmed that a chick *Dbx1* 3.5-kb CRM has the potential to recapitulate *Dbx1* expression in the developing chick spinal cord (Fig. S7C,D). Thus, one possible scenario is that the gene regulatory mechanisms for the embryonic spinal cord might have been co-opted to recruit *Dbx1* expression in the VP of a common ancestor of amniotes, and further functional modifications have occurred after the divergence of mammalian and non-mammalian (sauropsid) lineages (Fig. 6). Recent studies indicated that misexpression of *Dbx1* in the developing chick pallium reduced the self-renewal of progenitors while accelerating neurogenesis and inducing reelin expression (Nomura et al., 2008; García-Moreno et al., 2018). Accordingly, we speculate that suppressive mechanisms for *Dbx1* expression in the reptilian and avian VP are prerequisite for sustaining progenitor pools that give rise to the sauropsid-specific dorsal ventricular ridge (Yamashita and Nomura, 2017). It has been proposed that conserved neurogenic programs in ancestral amniotes have been secondarily modified to establish spatially biased neurogenesis in the developing avian pallium (Suzuki and Hirata, 2013). Our results provide mechanistic insight into ancestral neurogenic programs and their functional modifications during amniote brain development and evolution.

## MATERIALS AND METHODS

### Animals

Fertilized chicken eggs were obtained from a local poultry farm (Yamagishi Farm, Japan) and incubated at 37°C. The stages of the chick embryo were determined according to Hamburger and Hamilton (1951). Pregnant wild-type mice (ICR background, 2-3 months) were purchased from Charles River. Heterozygous *Small eye* (*Sey*) *Pax6* mutant mice (C57BL6/J background) maintained at Tohoku University were intercrossed to obtain homozygous *Sey* embryos. All animal experiments were approved by the Committee of the Kyoto Prefectural University of Medicine (M23-272) and Tohoku University Graduate School of Medicine (#2013-334).

### Immunohistochemistry and *in situ* hybridization

Brains were fixed in 4% paraformaldehyde in PBS at 4°C overnight, and then cryoprotected in a 20% sucrose solution and embedded in Tissue-Tek (Sakura). For immunohistochemistry, frozen sections (18 µm) were sliced with a cryostat (CM1850, Leica) and incubated with primary antibodies: anti-Pax6 (rabbit polyclonal, 1:500, PD022, MBL), anti-Gsh2 (rabbit polyclonal, 1:500, ABN162, Merck Millipore), anti-Tbr2 (rabbit polyclonal, 1:500, ab23345, Abcam; rabbit polyclonal, 1:500, HPA028896, Atlas Antibody; chicken polyclonal, 1:500, AB15894, Merck Millipore), anti-Tbr1 (chicken polyclonal, 1:500, AB2261, Merck Millipore), anti-GFP (rabbit polyclonal, 1:500, A11122, Thermo Fisher Scientific; rat monoclonal, 1:500, GF090R 04404-84, Nacalai Tesque), anti-GABA (rabbit monoclonal, 1:500, A2052, Sigma), anti-cleaved caspase 3 (rabbit polyclonal, 1:500, D175, Cell Signaling) and anti-Dbx1 [rabbit polyclonal, 1:2000, a gift from Dr Shirasaki (Inamata and Shirasaki, 2014)] antibodies. After washing in Tris-buffered saline containing 0.01% Tween 20, the sections were incubated with secondary antibodies: Alexa Fluor 488-, 594- and 633-conjugated anti-rabbit, mouse and rat antibodies (all at 1:500, Thermo Fisher Scientific). Nuclear staining was performed with Hoechst 33258. For Tbr2 staining, heat-mediated antigen retrieval was performed for 20 min at 70°C with HistoVT One (Nacalai Tesque) before incubation with the primary antibody. The sections were analyzed using a fluorescence microscope (BX51, Olympus) equipped with a cooled CCD system (DP71, Olympus) and using a laser-scanning confocal microscope (FV1000D, Olympus).

*In situ* hybridization against chick *Dbx1* was performed as previously described (Nomura et al., 2008). A digoxigenin (DIG)-labeled cRNA probe was synthetized by *in vitro* transcription from chick *Dbx1* cDNA that was subcloned into the *p3T* vector (Molecular Biotechnology). After the color reaction, images were analyzed using a fluorescence microscope (BX51, Olympus).

### *In vivo* genome editing of the chicken *Pax6* gene

Design and validation of CRISPR/Cas9-mediated *in vivo* gene targeting were performed as described (Shinmyo et al., 2016). For plasmid construction, we designed sgRNAs for the chicken *Pax6* gene using CHOPCHOP software (http://chopchop.cbu.uib.no/) (Montague et al., 2014), and three sgRNAs targeting the sequences of exon 5, 7 and 8 of *Pax6* were selected and cloned into *pX330-U6-Chimeric_BB-CBh-hSpCas9* (a gift from Feng Zhang, Addgene plasmid #42230; Cong et al., 2013). The primers used for plasmid construction are listed in Table S3. DNA solution (0.1 µl) containing *pX330* (2 µg/µl) and *pCAX-GFP* (0.5 µg/µl) was electroporated into the developing chick pallium. To validate insertions/deletions (indels) of target sequences, the GFP-positive region of the electroporated sample was dissected under a fluorescence microscope (SZX7, Olympus) 36 h after *in ovo* electroporation, and genomic DNA isolated using the DNeasy Blood & Tissue Kit (Qiagen). The target sequence of the sgRNA was amplified by PCR with KOD FX Neo polymerase (TOYOBO) and the following primers: 5′-TGTGGTTTTCTGTCCGCTTCCCT-3′ (forward) and 5′-CTGGGGATGACCGCGTCGTT-3′ (reverse). The PCR amplicons were used for the construction of a library for Illumina sequencing with the KAPA Hyper Prep Kit (Kapa Biosystems) and were sequenced using an Illumina Miseq sequencer. Indel analysis was performed by mapping the reads to the chick reference genome sequence (*Gallus gallus* chromosome 5; accession no. NC 006092.4). Under our experimental conditions, it took at least 48 h to detect phenotypes by introduction of sgRNAs; to examine rapid changes in gene expression or cellular dynamics by manipulation of Pax6, we utilized the expression vectors for *Pax6* or *Pax6-EnR*.

### *In ovo* and *in utero* electroporation

*In ovo* electroporation of the developing chick and *in utero* electroporation of the developing mouse neocortex were performed as described (Nomura et al., 2013). Briefly, ∼0.1 µl DNA solution was injected into the lateral ventricle of each embryo using a glass needle. Then, needle-type electrodes (CUY200S, BEX) were placed on the embryo head, and square electric pulses (28-32V, 50 ms, 3-4 times) were applied with a pulse generator (CUY21 EDITII, BEX). Electroporated chick embryos were placed in incubators at 37°C. To prepare the DNA solution, various expression vectors, including *pCAX-GFP*, *pX330-gRNAs*, *pCAX-Pax6*, *pMIWIII-Pax6-EnR*, were dissolved at 0.5-2.5 µg/µl in PBS containing 0.05% Fast Green.

### Clonal analysis

For clonal analysis of chick neural progenitors, *Cytbow* and *Nucbow* vectors, self-excision Cre expression vector (*pSE-Cre*) and a transposase expression vector (*pBase*) were mixed with a *pCAG* empty vector or *pCAX-Pax6*, as previously reported (Loulier et al., 2014), and electroporated into the E4 chick dorsal pallium. Fluorescent images of brain sections were captured by a laser-scanning confocal microscope (FV1000). Labeled cells with the same combination of fluorescent proteins in side-by-side serial sections were regarded as clonal siblings. We chose rare combinations of fluorescent proteins to avoid misinterpreting the clonal relationships of labeled cells.

### Quantitative real-time PCR (qRT-PCR)

After electroporation of *pCAX-Pax6* and/or *pCAX-GFP*, the GFP-positive chick pallial region was manually dissected under the fluorescence microscope, and total RNA was extracted using the RNeasy Mini Kit (Qiagen), and cDNA was synthesized using random primers with reverse transcriptase (ReverTra Ace, TOYOBO). qPCR was performed on a Light Cycler Nano (Roche) with THUNDERBIRD SYBR qPCR Mix (TOYOBO) according to the manufacturers' protocols. Gene expression levels were normalized to those of amplification of β-actin. Primer sequences for qPCR (Table S3) were designed by primer-BLAST (NCBI). qPCR was carried out on three independent samples with two technical replicates.

### Labeling of S-phase cells

To detect cells undergoing S-phase, 5-ethynyl-2′-deoxyuridine (EdU; 10 mg/ml, Thermo Fisher Scientific) was injected into the lateral ventricle in developing chick embryos (0.1 µl) and the peritoneum in pregnant mice (100 mg/kg) 1 h before fixation. EdU detection was with the Click-iT Plus Kit (Thermo Fisher Scientific).

### Luciferase reporter assay

To quantify the activity of Notch signaling, the vectors *p4xCSL-firefly luciferase* (Addgene #41726), *pRL-SV40* (Promega) and *pCAX-GFP* were co-electroporated into the dissociated neural progenitors using electroporation cuvettes (SE-202, BEX). After electroporation, neural progenitors were cultured as floating cell aggregates for 24 h in Neurobasal medium supplemented with GlutaMAX, B27 supplement (Thermo Fisher Scientific) and FGF2 (10 ng/ml), as previously reported (Yamashita et al., 2017). To examine the transcriptional activity of mouse and chicken *Dbx1* 3.5-kb CRMs, HEK293T cells (RIKEN BRC; contamination has been checked routinely) were transfected with *pGL3-promoter* vectors (Promega) containing mouse or chicken *Dbx1* 3.5-kb CRM together with *pRL-SV40* and *pCAX-mouse Pax6* vectors using Lipofectamine 2000 (Thermo Fisher Scientific). Luciferase reporter activity was examined with the Dual-Luciferase Reporter Assay System (Promega). Chemical luminescence was analyzed with a luminometer (GENE LIGHT GL210A, Microtec). All firefly luciferase values were normalized to *Renilla* luciferase activities to quantify relative luciferase units. Each experiment was carried out in four biological replicates.

### RNA-seq data analysis

After electroporation of *pCAX-Pax6* and/or *pCAX-GFP*, GFP-positive chick pallial regions were manually dissected under a fluorescence microscope. Total RNA was extracted from dissected tissues with the RNeasy Plus Universal Kit (Qiagen) according to the manufacturer's instructions. RNA quality was assessed using a NanoDrop 1000 spectrophotometer (Thermo Fisher Scientific) and an Agilent 2100 Bioanalyzer (Agilent Laboratories). The cDNA library was constructed using the TruSeq Stranded Total RNA Library Prep Kit (Illumina) and paired-end sequencing was performed with a HiSeq 2500 (Illumina). Sequencing data were mapped to a reference genome sequence (Galgal4; retrieved from the Ensembl genome browser database) and quantified by differential gene expression analyses.

### Isolation of mouse and chicken *Dbx1* 3.5-kb CRMs

The mouse *Dbx1* 3.5-kb CRM located 2.7 kb upstream of the transcription start site was isolated from C3H mouse genomic DNA and cloned into *pGL3-promoter* (Promega) and *pTAL-d2GFP* (Clontech) vectors. The chicken *Dbx1* 3.5-kb CRM was isolated from a BAC clone (CH261-63F7, BAC PAC Resources) based on chicken genomic information (Gallus_gallus-5.0). The primer sequences for PCR amplification of mouse and chick *Dbx1* 3.5-kb CRM are listed in Table S2.

### Chromatin immunoprecipitation assay

The transient ChIP assay was performed as previously described (Lavrrar and Farnham, 2004). Briefly, HEK293T cells were transfected with *pCAG-chick Pax6* and *pGL3-chick Dbx1* *3.5-kb CRM* using Lipofectamine 2000. At 24 h after transfection, cells were cross-linked with formaldehyde for 10 min and then harvested to isolate nuclear extracts. ChIP was carried out with the Simple ChIP Plus Kit (Cell Signaling Technology) according to the manufacturer's protocol. Anti-Pax6 antibody (MBL, 1:100), anti-IgG and anti-Histone H3 antibodies (Cell Signaling Technology, 1:250 and 1:50, respectively) were used for immunoprecipitation. Genomic fragments containing putative Pax6 binding sites (BS1-2, BS3-4, BS5-6, and BS7) in the chick *Dbx1* 3.5-kb CRM were amplified by qPCR with specific primers (Table S2).

### Quantification and statistical analyses

Cell numbers were quantified by image capture with a laser-scanning confocal microscope (FV1000D, Olympus) and analysis with ImageJ (NIH) software. Three representative sections of each sample were selected for cell counting. All quantitative data were obtained from six samples and are presented as mean±s.e.m. The variances of each data point were checked by *F*-test, and the statistical significance of mean values was calculated using two-tailed Student's *t*-test, Welch's *t*-test or Dunnett's multiple comparison test.

## Supplementary Material

Supplementary information

Supplementary information
