# Supplementary material for: Conserved and divergent functions of Pax6 underlie species-specific neurogenic patterns in the developing amniote brain
Source: Development. 2018 Apr 16;145(8):dev159764. doi: 10.1242/dev.159764 (PMC5964652; doi:10.1242/dev.159764)
Supplement: Supplementary information [file develop-145-159764-s1.pdf]

Supplementary Information

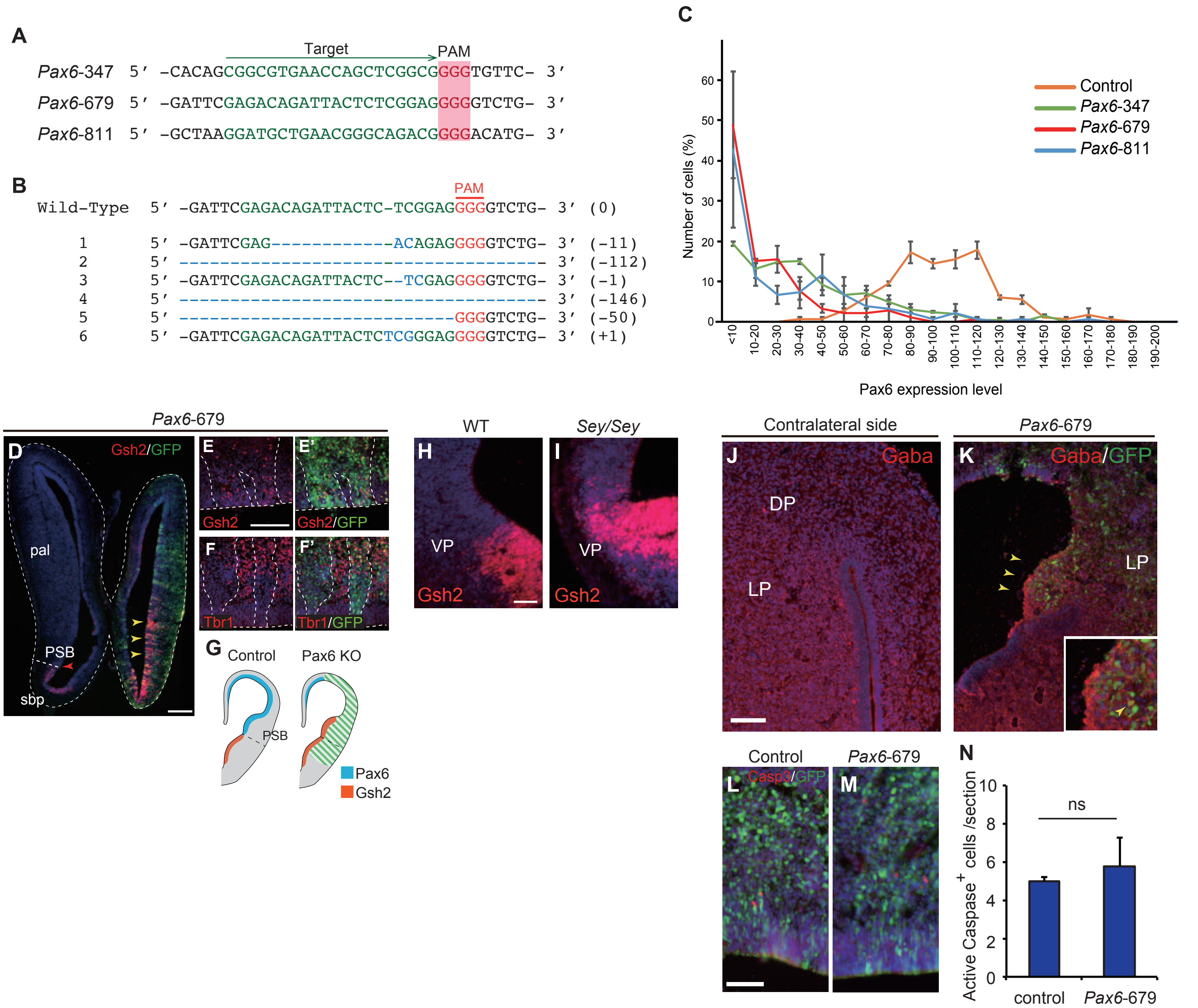

Fig. S1 (related to Fig. 1). CRISPR/Cas9-mediated targeting of the *Pax6* gene in the developing chick pallium.

(A) Target sequences of single-guide RNAs (sgRNA) (green) upstream of the PAM sequence (red) in the chick *Pax6* gene. (B) Representative mutant sequences of Pax6 after electroporation of *pX330-Pax6*-679. Indels are indicated in blue. Target sequences of sgRNA and PAM sequences are indicated as green and red, respectively. (C) The expression level of Pax6 in the developing chick pallium after electroporation of *pX330-Pax6* vectors. The histogram shows the signal intensity of Pax6 in samples transfected with control vector or *pX330*-Pax6 vectors (mean ± s.e., n=4 animals in each group). (D, E, E', F, F') Ectopic expression of Gsh2 and reduced Tbr1 expression in the E6 chick pallium transfected with *pX330-Pax6*-679. (G) Schematic illustration of the phenotype of the *Pax6*-deleted chick pallium. The area shown with a green oblique line indicates the electroporated region. (H, I) Gsh2 expression in E12.5 wild-type (H) and *Small eye (Sey/Sey)* (I) mice. In *Sey/Sey*, ectopic Gsh2 expression is evident at the VP. (J, K) Accumulation of Gaba-positive cells in the ventricular zone of E10 chick pallium transfected with *pX330-Pax6*-679. (L-N) Active caspase 3-positive cells in brains transfected with control and *pX330-Pax6*-679 vectors. Scale bars: 200 µm (D, H, J), 50 µm (E, L).

Development • Supplementary information

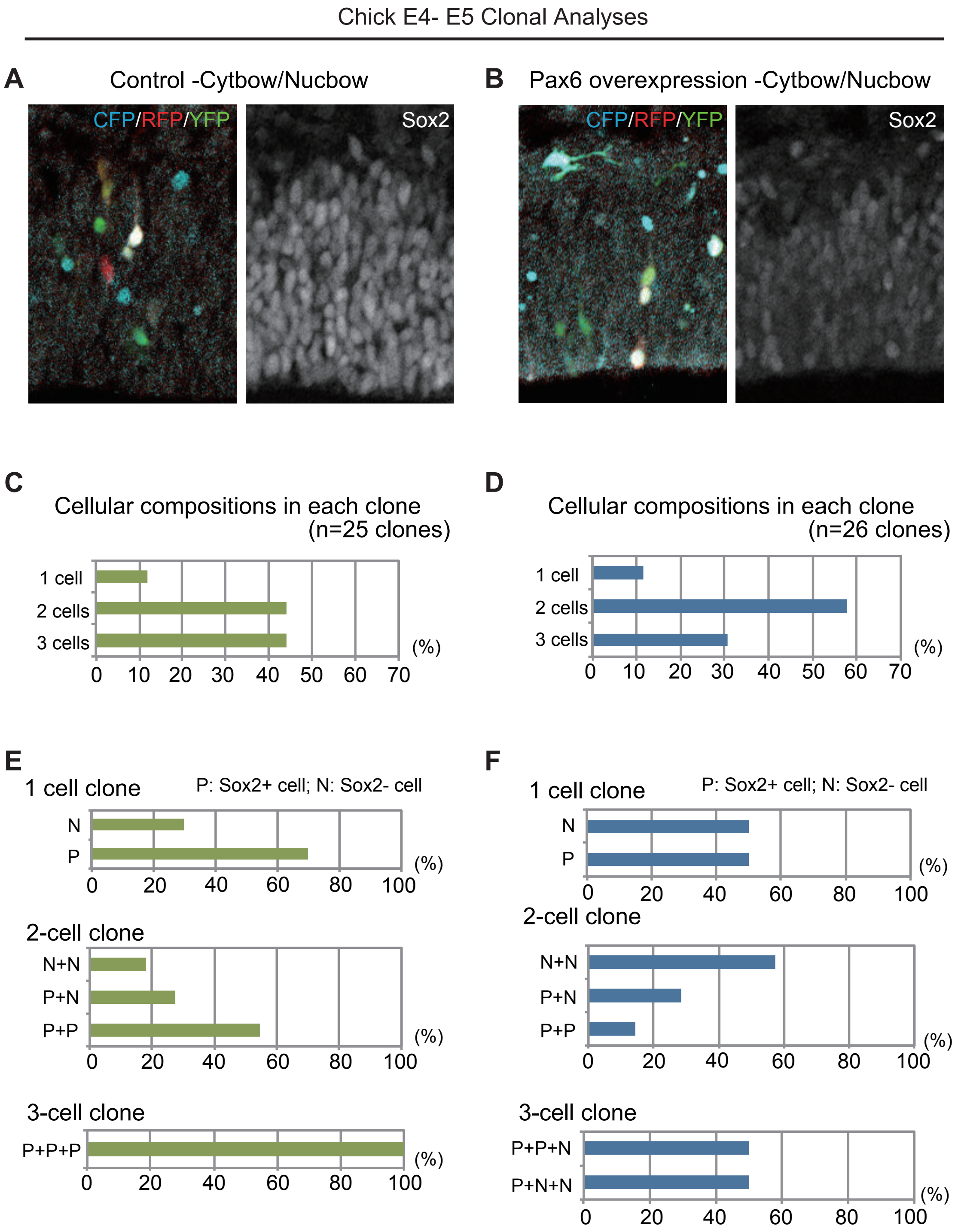

Fig. S2 (related to Fig. 2). High-dose Pax6 increases neurogenic divisions in the developing chick pallium.

(A, B) Distributions of clonally related cells in E5 chick pallium transfected with control and *Pax6* expression vectors. Clonal siblings are labeled by co-electroporation of *Cytobow/Nucbow* and the self-excision Cre expression vector. (C-F) Cell compositions in each clone in control (C, E) and *Pax6*-overexpressed brains (D, F). Progenitors (P) and non-progenitors (N) are distinguished by Sox2 expression. High-dose Pax6 decreases the proportion of progenitors and increases non-progenitors in individual clones. n=2 brains for each case.

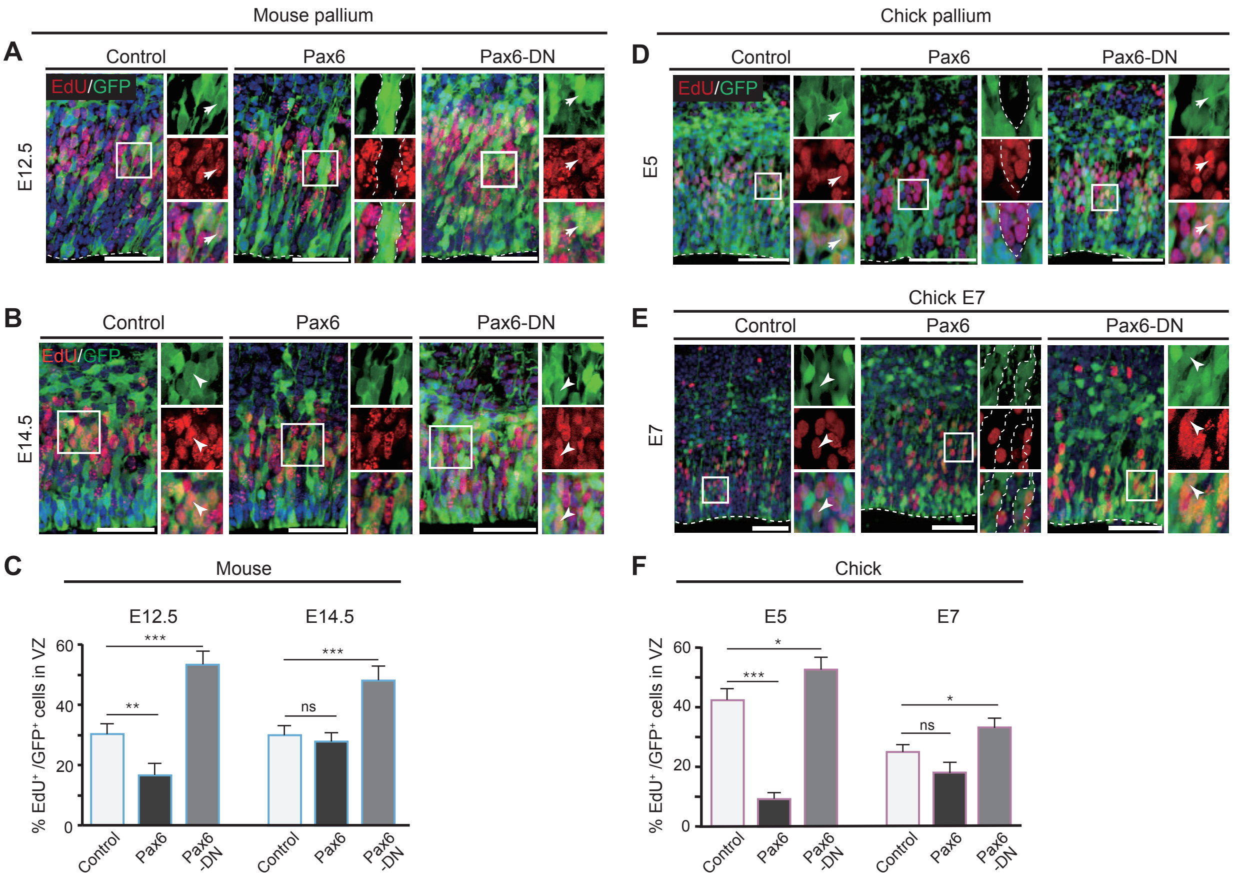

Fig. S3 (related to Fig. 2). Pax6-dependent negative regulation of progenitor proliferation in the developing mouse and chick dorsal pallium.

(A, B, D, E) EdU-positive cells in the E12.5 (A) and E14.5 (B) mouse and E5 (D) and E7 (E) chick dorsal pallium after electroporation of control (GFP), *Pax6* or *Pax6-DN* vectors. Insets show representative EdU-positive cells in GFP+ transfected cells (white arrowheads). (C, F) The proportion of EdU-positive cells in GFP-positive cells in the VZ of mouse (C) and chick (F) dorsal pallium. Data indicate the mean ± s.e., n=6 for each case, Student's *t*-test, $*P < 0.05$, $**P < 0.01$, $***P < 0.005$. Scale bars: 50 µm.

Development • Supplementary information

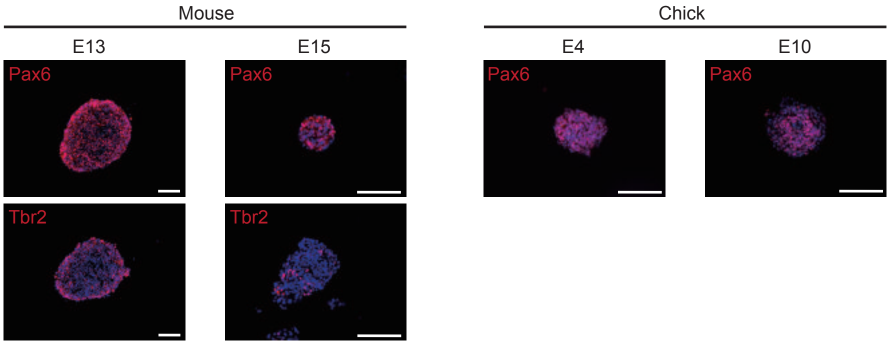

Fig. S4 (related to Fig. 3). Pax6 and Tbr2 expression in mouse and chick neurospheres. Immunohistochemstry of neurospheres derived from the E13 or E15 mouse neocortex and the E4 or E10 chick pallium with anti-Pax6 or –Tbr2 antibodies. Scale bar: 100 µm.

Development • Supplementary information

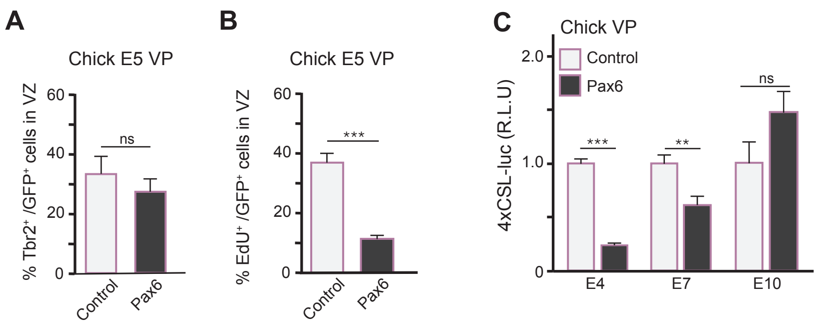

Fig. S5 (related to Fig. 3). Pax6 gain-of-function analysis of chick ventral pallial progenitors.

(A, B) Tbr2- and EdU-positive cells in the E5 chick ventral pallium after electroporation of control (GFP) or Pax6 vectors. (C) Pax6-dependent changes in Notch reporter (*p4xCSL*-luciferase) activity in neuronal progenitors from the E4, E7 or E10 chick ventral pallium. Data indicate the represented mean ± s.e., at least n=3 for each case, Student's *t*-test, $*P < 0.05$, $**P < 0.01$.

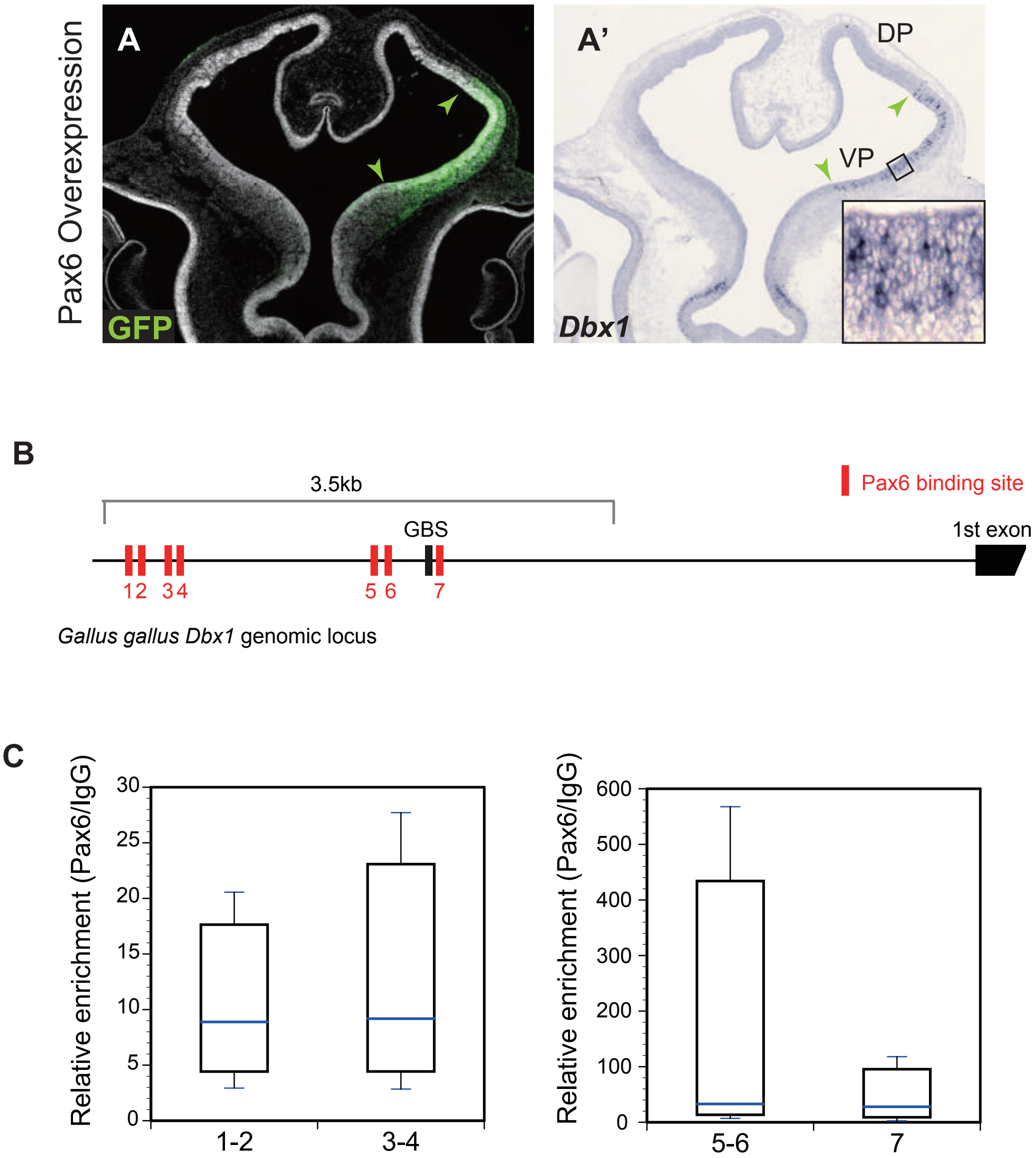

Fig. S6 (related to Fig. 4). Pax6-dependent activation of *Dbx1* and physical interaction of Pax6 on *Dbx1* 3.5-kb CRM

(A) Electroporation of Pax6 induces *Dbx1* expression in the developing chick ventral pallium (VP). (B) Distributions of putative Pax6 binding sites in chick *Dbx1* 3.5-kb CRM. GBS: Gli-binding site reported previously (Oosterveen et al., 2012). (C) ChIP-qPCR demonstrating relative enrichment of Pax6 binding sites (1-2, 3-4, 5-6 and 7) after immunoprecipitation with anti-Pax6 antibody.

Development • Supplementary information

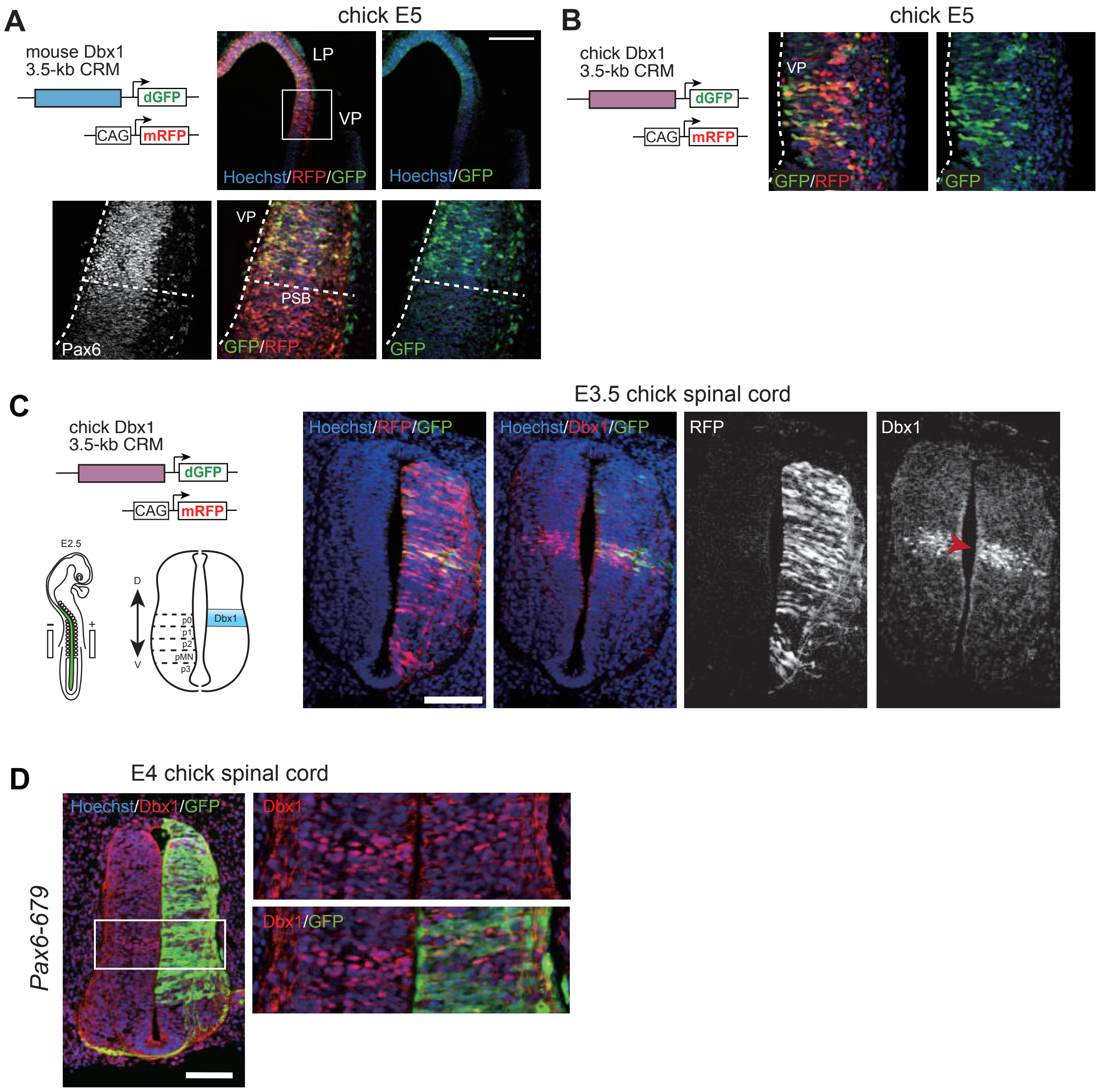

Fig. S7 (related to Fig. 5). Conserved enhancer activity of mouse and chick *Dbx1* 3.5-kb CRM in the developing chick pallium and spinal cord.

(A) dGFP expression driven by mouse (A) or chick *Dbx1* 3.5-kb CRM (B) in the developing chick pallium (E5). (C) Electroporation of chick *Dbx1* 3.5 kb-CRM reporter vector and *pCAG-mRFP* vector into the E2.5 developing chick spinal cord. The expression pattern of dGFP is consistent with endogenous Dbx1 expression in the developing spinal cord. (D) Electroporation of *px330-Pax6-679* down-regulates Dbx1 in the spinal cord. Scale bars: 100 µm (A), 50 µm (C). Scale bars: 100 µm (A), 50 µm (C, D).

Development • Supplementary information

test_id	gene_id	gene	locus	sample_1	sample_2	status	value_1	value_2	log2(fold_chang	test_stat	p_value	q_value	significant
ENSGALG00000003965	ENSGALG00000003965	DBX1	5:2045882-2049582	control:Ir2866_Ir5275_Ir5276	treatment:Ir2867_Ir5273_Ir5274	OK	0.128984	16.3228	6.98355	6.42028	0.0002	0.0383213	yes
ENSGALG00000012941	ENSGALG00000012941	CDH12	2:73046217-73413333	control:Ir2866_Ir5275_Ir5276	treatment:Ir2867_Ir5273_Ir5274	OK	0.168492	3.4192	4.34291	4.43095	5.00E-05	0.0135907	yes
ENSGALG00000017485	ENSGALG00000017485	TLR1LA	4:69131052-69133509	control:Ir2866_Ir5275_Ir5276	treatment:Ir2867_Ir5273_Ir5274	OK	0.20848	3.80424	4.18963	3.79324	5.00E-05	0.0135907	yes
ENSGALG00000001115	ENSGALG00000001115	MMEL1	21:1387459-1411822	control:Ir2866_Ir5275_Ir5276	treatment:Ir2867_Ir5273_Ir5274	OK	0.216831	3.30088	3.92821	4.72478	5.00E-05	0.0135907	yes
ENSGALG00000005887	ENSGALG00000005887	PXDNL	20:9382941-9424831	control:Ir2866_Ir5275_Ir5276	treatment:Ir2867_Ir5273_Ir5274	OK	0.123655	1.74019	3.81485	3.94011	5.00E-05	0.0135907	yes
ENSGALG00000016138	ENSGALG00000016138	DSCAM	1:107601009-107978889	control:Ir2866_Ir5275_Ir5276	treatment:Ir2867_Ir5273_Ir5274	OK	0.133329	1.8734	3.81259	4.10264	5.00E-05	0.0135907	yes
ENSGALG00000017926	ENSGALG00000017926	7SK	3:87768166-87768495	control:Ir2866_Ir5275_Ir5276	treatment:Ir2867_Ir5273_Ir5274	OK	20.5253	248.4	3.59719	4.61408	5.00E-05	0.0135907	yes
ENSGALG00000003488	ENSGALG00000003488	PTPRT	20:3189961-3615919	control:Ir2866_Ir5275_Ir5276	treatment:Ir2867_Ir5273_Ir5274	OK	0.205108	2.11757	3.36795	3.56082	5.00E-05	0.0135907	yes
ENSGALG00000016448	ENSGALG00000016448	KCNF1	3:96578937-96581015	control:Ir2866_Ir5275_Ir5276	treatment:Ir2867_Ir5273_Ir5274	OK	0.226339	2.28174	3.33357	3.13143	0.0002	0.0383213	yes
ENSGALG00000009737	ENSGALG00000009737	tac1	2:24773217-24779911	control:Ir2866_Ir5275_Ir5276	treatment:Ir2867_Ir5273_Ir5274	OK	1.20682	11.9954	3.31319	4.00289	5.00E-05	0.0135907	yes
ENSGALG00000003644	ENSGALG00000003644	-	10:3925575-3927300	control:Ir2866_Ir5275_Ir5276	treatment:Ir2867_Ir5273_Ir5274	OK	0.552565	4.57903	3.05083	3.24835	5.00E-05	0.0135907	yes
ENSGALG00000005246	ENSGALG00000005246	PAX3	9:7357517-7422566	control:Ir2866_Ir5275_Ir5276	treatment:Ir2867_Ir5273_Ir5274	OK	0.281772	2.32742	3.04613	3.08842	0.0002	0.0383213	yes
ENSGALG00000027381	ENSGALG00000027381	OLIG3	AADN03015019.1:920-27	control:Ir2866_Ir5275_Ir5276	treatment:Ir2867_Ir5273_Ir5274	OK	1.36112	10.9275	3.00509	3.34019	5.00E-05	0.0135907	yes
ENSGALG00000012027	ENSGALG00000012027	SPON1	5:5838065-6002800	control:Ir2866_Ir5275_Ir5276	treatment:Ir2867_Ir5273_Ir5274	OK	2.33476	17.0096	2.865	5.03756	5.00E-05	0.0135907	yes
ENSGALG00000006003	ENSGALG00000006003	-	13:13158122-13161628	control:Ir2866_Ir5275_Ir5276	treatment:Ir2867_Ir5273_Ir5274	OK	0.336353	2.30225	2.77499	2.89816	0.00015	0.0337154	yes
ENSGALG00000002864	ENSGALG00000002864	LRRTM3	6:6129270-6562977	control:Ir2866_Ir5275_Ir5276	treatment:Ir2867_Ir5273_Ir5274	OK	0.484915	2.96387	2.61168	3.23203	5.00E-05	0.0135907	yes
ENSGALG00000027720	ENSGALG00000027720	OTX2	5:55680635-55688787	control:Ir2866_Ir5275_Ir5276	treatment:Ir2867_Ir5273_Ir5274	OK	1.08184	6.55973	2.60015	3.01196	5.00E-05	0.0135907	yes
ENSGALG00000005967	ENSGALG00000005967	-	5:9538612-9582720	control:Ir2866_Ir5275_Ir5276	treatment:Ir2867_Ir5273_Ir5274	OK	3.06274	17.8172	2.54038	3.51923	5.00E-05	0.0135907	yes
ENSGALG00000010133	ENSGALG00000010133	PTCH2	8:19696866-19712815	control:Ir2866_Ir5275_Ir5276	treatment:Ir2867_Ir5273_Ir5274	OK	1.10459	6.40092	2.53477	3.6027	5.00E-05	0.0135907	yes
ENSGALG00000013624	ENSGALG00000013624	FAM65B	2:90153585-90209672	control:Ir2866_Ir5275_Ir5276	treatment:Ir2867_Ir5273_Ir5274	OK	0.944413	5.28567	2.4846	3.31535	5.00E-05	0.0135907	yes
ENSGALG00000016035	ENSGALG00000016035	GFRA4	4:88844983-88914622	control:Ir2866_Ir5275_Ir5276	treatment:Ir2867_Ir5273_Ir5274	OK	0.500423	2.77634	2.47196	2.86013	5.00E-05	0.0135907	yes
ENSGALG00000005519	ENSGALG00000005519	TLL2	6:16142743-16223667	control:Ir2866_Ir5275_Ir5276	treatment:Ir2867_Ir5273_Ir5274	OK	1.12504	5.26563	2.22663	3.06067	5.00E-05	0.0135907	yes
ENSGALG00000003541	ENSGALG00000003541	SLC32A1	20:4000629-4004091	control:Ir2866_Ir5275_Ir5276	treatment:Ir2867_Ir5273_Ir5274	OK	1.09842	4.83769	2.13889	2.56155	0.00015	0.0337154	yes
ENSGALG00000015419	ENSGALG00000015419	PENK	2:111076868-111081312	control:Ir2866_Ir5275_Ir5276	treatment:Ir2867_Ir5273_Ir5274	OK	3.87722	15.9351	2.03911	3.17814	5.00E-05	0.0135907	yes
ENSGALG00000010929	ENSGALG00000010929	SPARCL1	4:45106287-45113208	control:Ir2866_Ir5275_Ir5276	treatment:Ir2867_Ir5273_Ir5274	OK	2.86536	11.6232	2.02022	2.96313	5.00E-05	0.0135907	yes
ENSGALG00000003895	ENSGALG00000003895	PRDM12	17:5956540-5962675	control:Ir2866_Ir5275_Ir5276	treatment:Ir2867_Ir5273_Ir5274	OK	2.79254	11.284	2.01463	2.42497	0.00025	0.046381	yes
ENSGALG00000029020	ENSGALG00000029020	PPAPDC1A	6:30060585-30109050	control:Ir2866_Ir5275_Ir5276	treatment:Ir2867_Ir5273_Ir5274	OK	0.742306	2.9119	1.97188	2.59308	5.00E-05	0.0135907	yes
ENSGALG00000016749	ENSGALG00000016749	CNGA3	1:131289231-131309321	control:Ir2866_Ir5275_Ir5276	treatment:Ir2867_Ir5273_Ir5274	OK	2.52865	9.79782	1.95409	2.76615	5.00E-05	0.0135907	yes
ENSGALG00000020626	ENSGALG00000020626	PQLC2L	9:22146849-22157886	control:Ir2866_Ir5275_Ir5276	treatment:Ir2867_Ir5273_Ir5274	OK	4.99205	19.041	1.9314	3.1923	5.00E-05	0.0135907	yes
ENSGALG00000007588	ENSGALG00000007588	GAD2	2:16147742-16180423	control:Ir2866_Ir5275_Ir5276	treatment:Ir2867_Ir5273_Ir5274	OK	1.43714	5.09325	1.82539	2.60885	5.00E-05	0.0135907	yes
ENSGALG00000007361	ENSGALG00000007361	SS	9:14279798-14281333	control:Ir2866_Ir5275_Ir5276	treatment:Ir2867_Ir5273_Ir5274	OK	19.346	65.7312	1.76454	3.03532	5.00E-05	0.0135907	yes
ENSGALG00000012505	ENSGALG00000012505	LRFN5	5:59384567-59393956	control:Ir2866_Ir5275_Ir5276	treatment:Ir2867_Ir5273_Ir5274	OK	1.86441	6.29176	1.75474	2.32654	0.00015	0.0337154	yes
ENSGALG00000009415	ENSGALG00000009415	SMOC1	5:26991939-27112082	control:Ir2866_Ir5275_Ir5276	treatment:Ir2867_Ir5273_Ir5274	OK	1.74413	5.64102	1.69345	2.5928	0.0001	0.0254087	yes
ENSGALG00000023886	ENSGALG00000023886	ZNF804A	7:1771380-1902480	control:Ir2866_Ir5275_Ir5276	treatment:Ir2867_Ir5273_Ir5274	OK	3.91656	12.5334	1.67812	3.02459	5.00E-05	0.0135907	yes
ENSGALG00000006514	ENSGALG00000006514	FSTL4	13:15562923-15746366	control:Ir2866_Ir5275_Ir5276	treatment:Ir2867_Ir5273_Ir5274	OK	1.93563	6.09762	1.65544	2.28464	0.0002	0.0383213	yes
ENSGALG00000026592	ENSGALG00000026592	-	1:100373555-100373705	control:Ir2866_Ir5275_Ir5276	treatment:Ir2867_Ir5273_Ir5274	OK	3255.21	10093.3	1.63257	3.02182	5.00E-05	0.0135907	yes
ENSGALG00000008263	ENSGALG00000008263	FAR-1	12:17814859-18073537	control:Ir2866_Ir5275_Ir5276	treatment:Ir2867_Ir5273_Ir5274	OK	1.5951	4.92462	1.62637	2.7054	5.00E-05	0.0135907	yes
ENSGALG00000016709	ENSGALG00000016709	RCAN2	3:109338624-109369202	control:Ir2866_Ir5275_Ir5276	treatment:Ir2867_Ir5273_Ir5274	OK	7.4497	22.8787	1.61875	2.32006	0.0002	0.0383213	yes
ENSGALG00000016602	ENSGALG00000016602	ARHGAP6	1:123651001-123765375	control:Ir2866_Ir5275_Ir5276	treatment:Ir2867_Ir5273_Ir5274	OK	1.14284	3.47969	1.60633	2.32984	0.00015	0.0337154	yes
ENSGALG00000000750	ENSGALG00000000750	CAPN5	1:192455766-192506610	control:Ir2866_Ir5275_Ir5276	treatment:Ir2867_Ir5273_Ir5274	OK	1.34418	4.06355	1.59602	2.52612	5.00E-05	0.0135907	yes
ENSGALG00000011406	ENSGALG00000011406	NTN4	1:45466329-45505575	control:Ir2866_Ir5275_Ir5276	treatment:Ir2867_Ir5273_Ir5274	OK	1.42902	4.20594	1.5574	2.45542	0.0002	0.0383213	yes
ENSGALG00000001768	ENSGALG00000001768	TENM2	13:4187412-4738485	control:Ir2866_Ir5275_Ir5276	treatment:Ir2867_Ir5273_Ir5274	OK	4.3589	12.4201	1.51064	3.4309	5.00E-05	0.0135907	yes
ENSGALG00000002958	ENSGALG00000002958	DNER	9:9405231-9525036	control:Ir2866_Ir5275_Ir5276	treatment:Ir2867_Ir5273_Ir5274	OK	4.48093	12.5484	1.48563	2.50228	0.0001	0.0254087	yes
ENSGALG00000005176	ENSGALG00000005176	SCG2	9:7713654-7715544	control:Ir2866_Ir5275_Ir5276	treatment:Ir2867_Ir5273_Ir5274	OK	14.6951	35.348	1.26629	2.51991	5.00E-05	0.0135907	yes
ENSGALG00000000317	ENSGALG00000000317	NEFM	22:953201-957709	control:Ir2866_Ir5275_Ir5276	treatment:Ir2867_Ir5273_Ir5274	OK	8.04458	18.8846	1.23112	2.46643	5.00E-05	0.0135907	yes
ENSGALG00000012670	ENSGALG00000012670	NRSN1	2:58022131-58024847	control:Ir2866_Ir5275_Ir5276	treatment:Ir2867_Ir5273_Ir5274	OK	10.1155	23.4877	1.21534	2.27506	0.0002	0.0383213	yes
ENSGALG00000003193	ENSGALG00000003193	CRABP-I	10:3197349-3210246	control:Ir2866_Ir5275_Ir5276	treatment:Ir2867_Ir5273_Ir5274	OK	66.4452	150.042	1.17513	2.65086	5.00E-05	0.0135907	yes
ENSGALG00000009452	ENSGALG00000009452	GALNT16	5:27313855-27374224	control:Ir2866_Ir5275_Ir5276	treatment:Ir2867_Ir5273_Ir5274	OK	6.9461	15.3849	1.14724	2.5118	5.00E-05	0.0135907	yes
ENSGALG00000012620	ENSGALG00000012620	PTCH1	Z:41628097-41693685	control:Ir2866_Ir5275_Ir5276	treatment:Ir2867_Ir5273_Ir5274	OK	5.28048	11.3834	1.10819	2.35799	0.0001	0.0254087	yes
ENSGALG00000006258	ENSGALG00000006258	SPOCK1	13:13906201-14163956	control:Ir2866_Ir5275_Ir5276	treatment:Ir2867_Ir5273_Ir5274	OK	14.2906	30.6141	1.09914	2.50776	5.00E-05	0.0135907	yes
ENSGALG00000013357	ENSGALG00000013357	MTCL1	2:98628114-98720357	control:Ir2866_Ir5275_Ir5276	treatment:Ir2867_Ir5273_Ir5274	OK	6.57621	13.6063	1.04895	2.35061	5.00E-05	0.0135907	yes
ENSGALG00000006837	ENSGALG00000006837	TSPAN4	5:14631768-14696703	control:Ir2866_Ir5275_Ir5276	treatment:Ir2867_Ir5273_Ir5274	OK	20.0313	40.3151	1.00907	2.22582	0.0002	0.0383213	yes
ENSGALG00000006498	ENSGALG00000006498	PTPRN2	2:8785741-9406733	control:Ir2866_Ir5275_Ir5276	treatment:Ir2867_Ir5273_Ir5274	OK	14.0255	25.9624	0.888378	2.10094	0.00025	0.046381	yes
ENSGALG00000001472	ENSGALG00000001472	JAM3	24:2508055-2515621	control:Ir2866_Ir5275_Ir5276	treatment:Ir2867_Ir5273_Ir5274	OK	40.3631	72.1941	0.838843	2.10472	0.00015	0.0337154	yes
ENSGALG00000005785	ENSGALG00000005785	-	W:22484-138725	control:Ir2866_Ir5275_Ir5276	treatment:Ir2867_Ir5273_Ir5274	OK	9.6203	4.27441	-1.17036	-2.35752	0.0002	0.0383213	yes
ENSGALG00000014366	ENSGALG00000014366	HNRPK	JH376210.1:13810-34765	control:Ir2866_Ir5275_Ir5276	treatment:Ir2867_Ir5273_Ir5274	OK	55.7702	23.725	-1.23309	-2.80719	5.00E-05	0.0135907	yes
ENSGALG00000006323	ENSGALG00000006323	LECT2	13:14532810-14537759	control:Ir2866_Ir5275_Ir5276	treatment:Ir2867_Ir5273_Ir5274	OK	8.67934	0.558908	-3.9569	-3.67994	0.00015	0.0337154	yes

Table S1. Genes that were up- or down-regulated by Pax6 overexpression in the developing chick pallum

```
mouse_Dbx1-3.5kb      ------------------------------------------------------------
chick                 CCTGTCTTCCCTAAAGAGTTTATACCTTCCCCAAAGAGTTTACTTCCTAAAGAGTTTATA 60

mouse_Dbx1-3.5kb      ------------------------------------------------------------
chick                 CCTGTTCTACATTCCAGTCATGAGAGCCATTCTGCCACGTCTCCATGATGCCAATTGCAT 120

mouse_Dbx1-3.5kb      ------------------------------------------------------------
chick                 CATAGCCCCATAGGCATGCACACCACGCACCTGTAACTCATCTTACTTGTTCTCCATGCT 180

mouse_Dbx1-3.5kb      ------------------------------------------------------------
chick                 CTCTGCGTTAGCATACAGGCATTTAAGTTGGGCCCCCATTAAGGCCTAATCAATTGGAGT 240

mouse_Dbx1-3.5kb      ------------------------------------------------------------
chick                 TCCCTTGTCCTTCACTTCAGATGTTCTCTTCCTGGCCTGTGGTCCTATTCCAGGACATCC 300

mouse_Dbx1-3.5kb      ---------------------CTGAGAAGGCTGGAAGAG-----------GGCATCAGAT 28
chick                 ATTATCGTCATCAGCCTCAAAGTGTGAGGAGTTGAAGACCACCTCCTCCCAGCATGAGCT 360
                                            ** ** *  * *****             **** ** *

mouse_Dbx1-3.5kb      TACGCTGGAGCTGGAGTTACAGACAGTTGTGTGCCAC-CTGATGTGGGTGCTAGGAATAG 87
chick                 TTTCCCCTGACTTCAGCTCTGATAGAGCAAATAACGTTCTCAGGCTAAAGCTACGGGGAA 420
                      *   *     **  ** *             *  *   ** * *     **** *   *

mouse_Dbx1-3.5kb      AA----CTCTGGTCCTCAGCAAGGGCAGC-ATACTCTCTTAACTGCTGAGCCATACCTCA 142
chick                 AAGCTTTTCTAAAATTGCCTTTACGCATTTGTCCTCTCCTTAGTCCATGACACTTCCATT 480
                      **     ***     *        ***    * ***** * * * *    *  * **

mouse_Dbx1-3.5kb      AATCTCCAGAACTGGTTTAATATTAACATGGCA---ACTGGCCAAGAG-----CCAGGTT 194
chick                 TGCTTTCAGAAAGCTGTAGACATCAGTGGAGGACTGATGGGACATGTATCTCCCCAGCCT 540
                          * *****     *  * ** *     * *   *  ** ** *       ****  *

mouse_Dbx1-3.5kb      GCTGTAGACCCCACAACATAGGACATAGACTTCTTATATGGAGCATG-AACAAAACTGTC 253
chick                 GATGGCTGGTGGAAAACA-GGGACAGCATTTCTTCCTGCTTGTCAGATAGCAGCACCATT 599
                      * **        * ****  *****     *  *  *      **   * **  **  *

mouse_Dbx1-3.5kb      TTCCAGGAAGGGCGGAGTGGTCTCCAGG----ATCTGTACCCAAGATACCAGTTCCC--C 307
chick                 CTCTGAGGAGGAACACACTTCCTACAGGCAAAACTCCCATCAAAAGTGTCTGGTCTTTGC 659
                       **   * ***          ** ****    *     * * **  *  * * **    *

mouse_Dbx1-3.5kb      CAGGATGACCTCTGCTGGGGTGCAAAAGGAGCAGCTGGTCCACACCTGGGTCCTGGGAGA 367
chick                 TATTTCAAAGAAATCTTACAAGTGCAAGTGAGATCTGAACCTCACC--AACTTTTCCTGG 717
                       *     *      **     *   ***    * ***  ** ****       *    *

mouse_Dbx1-3.5kb      TCAGAGAAGGGTGAACAGATATCTGTCTGTGGACA-AAAGCAGCCATTTTGGTAAGGAAG 426
chick                 TGATAGAATGGAAGAGAGACTTTCATCTCCAAATACAGGGAAGCCACAACATCT--GCAA 775
                      * * **** **   * ***  *   ***    * * *  * *****          * *

mouse_Dbx1-3.5kb      GGCAGCCTTTTCACCCAGCCAACTTCACCTTCACCTAAGCCTCT--CTGAATGAGGAAGA 484
chick                 AGCAAACCCAGAAGAATGGCAGCCCCGCTCCAGTCTCTGCAATAAGCCGAGTGTTTCCTA 835
                       ***  *     *    * ** *  * *      **  **      * ** **      *

mouse_Dbx1-3.5kb      GACAGAAAATCAATAGGTCGAATTTGGGATAAGAAAGGGGGAGCGGGGGGGGGGGGGAGA 544
chick                 AAGAGAACAACTCTTACTCACACTCTGCAAAGAATTGGTGGTTCTGAGTCCAGAGCTATG 895
                       * **** * *  *   **  * *  * * *  *  ** **  * * *    * *  *

mouse_Dbx1-3.5kb      CTTGGAAAGAATGAAGCTGCAGCGTGAATGAAGCAACTGAGGCTCATGACAAGGATGGTG 604
chick                 GACTCGGATCACGTTGCAAAACCTGAAGAGGTGTTAGTGGTACACAAGACTACAAAGCTA 955
                             *  * *  **   * *   *  *  *  * **   * ** *** *  * * *

mouse_Dbx1-3.5kb      GCTAA-----GGAGGAGACCCTCCTTTCTTCCACTGGTGTCCAGGC--AATACCTCCTGG 657
chick                 TTCATTCCTTGGCCACAACTATTCCATCCTCCATCATTTTCTCTGTCTGAAACATCTCAA 1015
                         *      **     **  * *  ** ****    * **   *    * ** **

mouse_Dbx1-3.5kb      TGGAGGGAGGGCCTAAGAGTAGCTTCAATGGGGCAGAAAGGGTCAGGATAGAACCTGAGG 717
chick                 GTCAGCAAAAAGCAGCCCACAAGGGCAAACAGAAACGTGGCTGCCTGCTTTCATCTGCTT 1075
                         **  *    *       *    ***   *  *    *   *  * *   * ***

mouse_Dbx1-3.5kb      GTTTG-TTCATCTCTGAGACCTGGGAGTGG-------GGAGAGCAAAATGTAGGCTTGGA 769
chick                 TTCCAATTTACATCAAGGAACTTAGAGCAGAACTCATGGACTGCATGGACCAGAGCTTTC 1135
                       *    ** *  **   ** **  ***  *       ***  ***      **   *

mouse_Dbx1-3.5kb      CAACCCTGTCCTCGCTGGC-CAATGTAC--AGGCCCCCAACAGGTC----TTGCTTCCCT 822
chick                 TGATTCATTTCTTATCGGCTCTATGAAGTGAAACCCCCTGCAGGACAAAGTTTCTTCTCT 1195
                        *  *  * **    *** * *** *   *  *****  **** *    ** **** **
```

```
mouse_Dbx1-3.5kb        CTTCCTGAAATACTTCTTTCTGGCATTCAT-TCTAGGCCCAAAGGCTCAGCTATTGGGGC 881
chick                   CTCTTCTTCAGTCAAAGCTCTGGAAATCAAATATATTGTATACTGCTTTTCCAAAAAGGC 1255
                        **       *  *     ***** * ***  * **      *  ***   * *    ***

mouse_Dbx1-3.5kb        CC-CAGAGGCAGCTGTGGCAACAGCAGGAGTGTTTTCCATTCCAAGAAGCTCAA------ 934
chick                   CAGCACATGTAGTCTCCTCCTCTGTGGATGCACTGCAGTTTACCAGAGACCTATGGATGA 1315
                        *  ** * * **      *  * *  *  *   *     ** * ***  *  *

mouse_Dbx1-3.5kb        -------AGTGGAAAGTTTTTTTTTT-------TTTTTTTTTTTTTTTTTTAAGAGCTC- 979
chick                   GTACAGCAGTGGGTAGGGCTGGTTCTGAAGAGATTTACCACTGCCATTGGCAAACGATCA 1375
                               *****  **   *  ** *       ***     *    **    **  * **

mouse_Dbx1-3.5kb        ------TATGAAACAACCTTTTATCTCACCCATGGATCCACTGAGGCTCGGGGGAAGAGG 1033
chick                   CCATTTGTGTTGCAGTCAGTCAGTAGGAGTAGAGTTGAAACCTGATTTGGGGTGGGGGG 1435
                              * **   **  *  * *        *  *  *  *    *  * ****   * **

mouse_Dbx1-3.5kb        ACTTTCTTTACAGAC-ACACCA---TAAGCGAACTCATGCAAACATGAGTTAAAAGGCAA 1089
chick                   AATCCTTTAATTCTCTGCACCGACTTTAGTGGAACCAGAGAAGTACATGTTGGA-GAAAG 1494
                        * *   ** *    *  ****    * ** * *  **   **  *   ***  * *  *

mouse_Dbx1-3.5kb        GAACTGGGCTGGTAAGGCGCTTGCCCCCAAGCCAGATACCCTGACTTCAATTCTCAGGAC 1149
chick                   TAAGGAGGAAGGTA--TCACTTTCCCTTGTCCTTGAGGAAATTAGGGCAAAGAATAAAGT 1552
                         **   **  ****   * *** ***      *  **      * *   ***     *

mouse_Dbx1-3.5kb        CCACCCATCTGGTAGGACAGAACCAACTCCTGCCAGTTGTCCTCTGACTTACACACACAC 1209
chick                   AC-TGTGTTGCACTCTGCAAACCCCAGCTCTCCAAGTCACACACAACCCAGTCTGTGAAA 1611
                         *     *         ** * ** *    ** * ***     * *   *          *

mouse_Dbx1-3.5kb        ACACACACACACAGAGAGAGAGAGAGAGAGAGAGAGAGAGAGAGAGAGAGAGAGAGAAAG 1269
chick                   GAAGCTGGCTTTAGGGAGGTTGTGCATTTGATTGTTTGTTATTAGCCAGATCAGTGGAG 1671
                          *         ** ***   * *      **   *   *      **   ***       **

mouse_Dbx1-3.5kb        AGAGAGAGAGAGAGACTATTTTTAAATTCAAAGATTAGAATTAGGCAATCGAGGAAAAGA 1329
chick                   A-AGAAAGAAGGAGGATTGTTCAACCCTGGAATATTACTATTTTAAAACAAGAGTGGAGG 1730
                        * *** ***  ***  *  **  *   *  ** ****  ***     **      *   **

mouse_Dbx1-3.5kb        GAAGGGAGTTTATCTTCAAGACTAAGAGTGTAAACTTTGAACTTAATTTCTCAGATAAGG 1389
chick                   TTGGGGAACAGATAAA-AGGATGAGGTGGGCAAAAATT--ATCATATCTGCAAGAAAGGG 1787
                           ****    **    * **  * * * * ***  **   *    ** *   *** * **

mouse_Dbx1-3.5kb        TGGTGGGGGGATGGGGGAGGTGATAGAAAG-GCACTGAAGCCTCGGCTCACCAA--GGTT 1446
chick                   TGCCAAGTAAACAGGCTAGGGAAGAGTTAGCAAAGCGTAAGCAAGACACAGGGACTAAAC 1847
                        **    *   *  **   ***  * **   **    *   *  *  *  * * **    *

mouse_Dbx1-3.5kb        TAATTTTGATCATTTCCAGTGGGCTCTGATGTACTGCCTAGCACTCTAGGGCTTTGCATC 1506
chick                   TAAAGTAAATTACTTCTGACAAGACGGGATCATCTGGTT-GCATTTCAGGCCATGCGCTC 1906
                        ***  *  ** * ***      *     ***    ***  * *** *  *** * *    **

mouse_Dbx1-3.5kb        CCTGGGAAGGAGGGGGGGGCTCAGGGGGACTACCAAGCCAGTACCACTGCACTGGGCTGC 1566
chick                   TCTGGGCTGGCCAGGATGAATGGTGAGGGAGAAGAAAGTAAC-TCTCCCCTCCAGGTCCC 1965
                         *****  **   **  *  *   *  **    *  **   *    *  *  *  **  *

mouse_Dbx1-3.5kb        CCTACAGAG----AGCCTTTTTCTTTGAATTAAAAAAAAAATTAAAAGGAAAAAGAAAAG 1622
chick                   TCTGCCAAGTCTGGGCCGCACGCTCTGTCCAAGATCCCTCTTTCTTTGCTCAGAGAGAGA 2025
                         ** *  **     ***     ** **    *  *       **     *   * *** *

mouse_Dbx1-3.5kb        AAAACACAAAAAAACACAACACACAGTTAACCACCTGTCAGGGCTGAGGATACAATAAGG 1682
chick                   GAGGGGGAGAGGAGAGAGAAACACAGTTAACCACCTGTCAGGGCTGAGGACACAATAAGG 2085
                         *     * *  *    *  ****************************** *********

mouse_Dbx1-3.5kb        GCCTTGGAAAAAGAGCTTTTCATGCATGCCTAATATATTTGTTCAGCTATTCACTTAATG 1742
chick                   CAGCCCCCAAAAGAGCTTTTCATGCATGCGTAATATATTTGTTCAGCTATTCACTTAATG 2145
                                ********************* ******************************

mouse_Dbx1-3.5kb        AAGCTGATCAATGTGATGGGAGACAATGGTCAAGTTAATCTATTTAGTAAAATGTTTTGA 1802
chick                   AAGCTGATAAATGTGATGCGAAACAATAGTCAAGTTAATCTATTTAGTAAAATGTTTTGA 2205
                        ******** ********* ** ***** ********************************

mouse_Dbx1-3.5kb        GACTTTCAGGCTATGAGAAGGAGGGGAGCCT--TTGGCGACATTTTCATTTAAATAACAC 1860
chick                   GACTCTCAGGCGATGTTAAGGAGGGTAGTGTGTTCAGGGACATTTTCATTTCAATA-CTC 2264
                        **** ****** ***  ******** **  *  *  * ************* ****  * *

mouse_Dbx1-3.5kb        TCCTCTCTCTTAGGCTGCACAAGGGGGGTTCAGGCAATCTATGTGAATATAATTTCTTATA 1920
chick                   TCATCTCTCCC---CTCTTAGGGGGGGTTCGAGCAACCTATGTGAATATAATTTCTTATA 2321
                        ** ******     **      *********  **** **********************

mouse_Dbx1-3.5kb        AACGTGTCTTTAATGGGTTTAATTCACCCTTGCCAGCTGTTTTGGGAGCTATTTTGTAAG 1980
chick                   AATGTGTCTTTAATGGGTTTAATTCACCCTTGCCAGCTGTTTTTTTAGCTATTTTGTAAG 2381
                        ** ****************************************   **************
```

Development • Supplementary information

```
mouse_Dbx1-3.5kb        AGCAAAACAAACGTTGCAGGCCTGGAAAGCTAGTTAAAATTAGTTTCGGTCAGCGAGGCC 2040
chick                   AGCAAAACAAACGTGCCAGGCTTGGAAAGCTAGTTAAAATTAGTTTCGGTCACTGGGGCC 2441
                        **************  ***** ******************************  * ****

mouse_Dbx1-3.5kb        AAGCTATATTTTCTAGAAACCATCTGCTTGATTGGCACAATAGGAGGATTTTCTAGGAAA 2100
chick                   AAGCTATATTTCCTGCAAACCATCTGCTTGATTGGCACAATAGAAGGATTTTCAAGAAAA 2501
                        *********** **  *************************** ********* ** ***

mouse_Dbx1-3.5kb        AAGAGGGGAGCGAGTGTGAAAGGAAGGGGGGCGTGCTAGAA--ACGGTTGGGGCCGCTGG 2158
chick                   AAAAAAAAAAAAAAAAAGGGCGGGGGGGGGGGGGGGGGGGGCGGAGGTTGTTGAATCTAG 2561
                        ** *    *   *    *   **  ****** * *   *      *****  *   ** *

mouse_Dbx1-3.5kb        TAGCTCCTTGGGCCTATTCTAAAGGTGAGCCCCTTAGCGTTATCTTAATAGAGGCTGGAG 2218
chick                   AAAAGCTTTGAGCGAACCAACAGTTACTGTTCCTTGTTCTGAATTC--TAAAGG-TGCAG 2618
                         *   * *** **  *     *      *  ****    * *  *   ** *** ** **

mouse_Dbx1-3.5kb        CTGTAGAAACGCTTTCTGGCTGTGCCTGGAGCACTCCTCCGTTGCCAGTCCAGACAGCAT 2278
chick                   CCTTAG----GTGTCGCAGTGCAGCCCCATTGAGGCTCCCGGCGCCCGGCCGGA--GCGG 2672
                        *  ***    *  *    *    ***      *  *  ***  *** * ** **  **

mouse_Dbx1-3.5kb        TCTCCTCCCGACCGGGAGAAGCCTGGGGTTGGGGGTGGGGATGGGTGGGTGCAAGTCCCT 2338
chick                   CCGTCCCCGG---GGGGAGCGCAGGCAGCACGGGACGGAGGCGCTTCGCTTCATCCCGCC 2729
                         *  * ** *   ***    **  *  *   ***  ** *  *  * * * **   * *

mouse_Dbx1-3.5kb        GTAGCCTCCTACTCAGGGAACTGCC--CGCCTCTTCTAGTGTACTTTCTCCATGTTCCAG 2396
chick                   CGGCCCTGGGCGGCCCCGCGCTGCCGCCGGCACTCCGGGAGC-CGGGCCCGGCGCTCCGT 2788
                            ***      *   *  *****  ** * ** *  * *  *   * *   * ***

mouse_Dbx1-3.5kb        TGCGGTGCGGAGGACCAAAGCCTGTGGGTTTGCAGCTTGCAAAAGGCAGCCTTAGCATCC 2456
chick                   GTCCGCGCTGGAAGTGGCGGCTCTCCCAATTTCA---TGCGGATTACCAACCCCGGCCCG 2845
                          * * ** *         **        ** **   ***  *   *   *   *   *

mouse_Dbx1-3.5kb        TGCAACCCTGAAGCTGGCACCACTTTCATTCCCTGAGAGCTGTGA-AGCTCCTCGCCAGG 2515
chick                   GGACACACGCAGACTGCCGCCGCCCCTCGGCCCCACCGCCGGCGTTAGTCCCCCCCCTTT 2905
                         *  ** *  *  *** * ** *       ***      * * *  **  ** * **

mouse_Dbx1-3.5kb        TGGCCCAAGTTTCCTTAGTCTAGACACTCCCCGGCTGGAGAGGTCTGTTGAGAGCCAGGA 2575
chick                   TTTTTCCCTCTTTTTTCGCGTAAGAA------AGCGAGTGAC-TTTTTAGAAATAAATTT 2958
                        *    *    **  ** *  **   *       **  * **  * * * ** *   *

mouse_Dbx1-3.5kb        GGTCCTAAAGGCACTTACTTGCCTAACCAAAAGGGACACTCAGCTTCTCTGGCTAGGGCC 2635
chick                   GAAGATAACGACATTAAAATG---AATGGCAGGAAGCACAAAACCTCGTGAGCATAGGTC 3015
                        *    *** * ** * *  **   **    * *   ***  * * **    **   ** *

mouse_Dbx1-3.5kb        TGGGGCTGGGGCTGGATCTGCAGCAGCCTGGTGCTGCAAAGCAGAGTAGGACCTGACCCC 2695
chick                   --------AAACGGCATTAGTCACAGGTTT-TATCCCGGGACATTAGAATTCCCTTCTCT 3066
                                   * * **  *   ***  *  *    *    **    *   **   * *

mouse_Dbx1-3.5kb        CTAGCCCGAGTCTCCTCTTATCTTTTTTCTTTGGTTTTGCTTTTTGAGACAGGGTCTTAC 2755
chick                   TAAGTGATCACCGCCTTTGAAAAGAAACTTCCAAAGGAGATCGCTGAAAATGAGATCCTT 3126
                          **       * *** * *         *        * *   *** *  * *

mouse_Dbx1-3.5kb        TTTGCAGACTTGGCCAGCCTGGAACTCACTGCCTAGAGTAGGCTGGTCTCGAACTCTCAG 2815
chick                   TCGTCTGATTTCTTGGTTC-----CTCGCGGCCTCTCGTCCCTTTCAATAGACGCCTC-- 3179
                        *   * ** **       *     *** * ****   **    *    * **   ***

mouse_Dbx1-3.5kb        AGATCCTCTTGCCTT--TACCAATTGGATGCCAGGATTAAAGGTGTGCACCTTCTTCCCT 2873
chick                   -GTCCCTTTTGCCGTGGTGCTTAAGGAATTCT----TTACGAGTGTGCTCAGACGCATCG 3234
                         *  *** ***** *  * *  *  * ** *     ***   ****** *   *    *

mouse_Dbx1-3.5kb        GGAAAGAGAAAGGCAGAAAGCTGAAGTGCGGTTTAAGAATGGGAATGTTTTCTCCGACAT 2933
chick                   ------------------------------------------------------------

mouse_Dbx1-3.5kb        AAACTTAAAGATAACGACGACTTTACAGTGAATGTCAGGACTCACAAAACCCCTCAAGCT 2993
chick                   ------------------------------------------------------------

mouse_Dbx1-3.5kb        CAGGTCAAATGGAATTAGTCACAGGACTTAGGCGCGGCCGCCATGCGCGTGGCCCTCGGT 3053
chick                   ------------------------------------------------------------

mouse_Dbx1-3.5kb        GGCCACGAGTTTGAAAAGGCGCGTTCAGAAATCACTTTAAAGTAAGATCCGTTCCTGGCT 3113
chick                   ------------------------------------------------------------

mouse_Dbx1-3.5kb        TTTGTTTTATTTTGTTTTGTTTTTTCCTCCCTTAGGATTTAATTTTGTTCTTTCAATGGC 3173
chick                   ------------------------------------------------------------

mouse_Dbx1-3.5kb        GGCCAGCCTTGCTTCCCCCACCTCCGCGAACCCTCACTTTCGCTCTTTGCCACCAGTGGA 3233
chick                   ------------------------------------------------------------
```

```
mouse_Dbx1-3.5kb      AAGGGTCTATGTGGATCATGAACGGGTAGACCTACCTTATAGTGAGTCAAGGTGGCCTCG 3293
chick                 ------------------------------------------------------------

mouse_Dbx1-3.5kb      TAAAAACAGAGCTTTAGATACGTTTGGATTGTGGAGAGCAAAACTGCCGGAGGATGAGGA 3353
chick                 ------------------------------------------------------------

mouse_Dbx1-3.5kb      GGGTAGCTAGCCTTCCAGGGCTGCAGAAACCACACCTCCACGGAAGTAACCTCGGGAGGC 3413
chick                 ------------------------------------------------------------

mouse_Dbx1-3.5kb      GGGACCTGGGAAGACCCACTGTGCTGTCTAATCTTTTCTTTCTTGGCAGAAACCTAGCG 3472
chick                 -----------------------------------------------------------
```

Table S2. Sequence comparison of mouse and chick Dbx1-3.5kb CRMs.

Pair-wise sequence alignment was performed by CLUSTALW. Asterisks represent conserved sequences. Putative Pax6-binding sites are represented in red (see also Figure S6B). A Gli-binding site (Oosterveen et al., 2012) is shown in green.

Development • Supplementary information

List of primers used for qPCR

Target	Forward primers	Reverse primers
beta-actin	CAGACATCAGGGTGTGATGGT	TCCTCAGGGGCTACTCTCAG
Ccnd1	CTTGGATGCTGGAGGTCTGC	CTGCGGTCAGAGGAATCGTT
Cdk6	GGCCTAATGATGTGGCCCTT	TCTTGGCTGGATTGAACGCT
$p27^{Kip1}$	GCCGACGATTCCTCTCCTCAAAA	ATCTTCCTGGCTTCACCGCC
Notch1	GAGCAGAGAGGGATGAAGCG	CACTGCTGCACTGGCACA
Dll1	TTCGGTCACTTCACCTGTGG	ACCCACTCTGCACTTGCATT
Dbx1	CAACCGAATTCCCAGCTATT	GGACAGTGGTTTGTCTGCAC

List of primers used for pX330 plasmid construction

	Forward primers	Reverse primers
pX330-*Pax6*-347	CACCGCGGCGTGAACCAGCTCGGCG	AAACCGCCGAGCTGGTTCACGCCGC
pX330-*Pax6*-679	CACCGAGACAGATTACTCTCGGAG	AAACCTCCGAGAGTAATCTGTCTC
pX330-*Pax6*-811	CACCGGATGCTGAACGGGCAGACG	AAACCGTCTGCCCGTTCAGCATCC

List of primers used for isolation of Dbx1-CRM

Target	Forward primers	Reverse primers
Mouse 3.5 kb-CRM	CTGAGAAGGCTGGAAGAG	CGCTAGGTTTCTGCCAAG
Chick 3.5 kb-CRM	CCTGTCTTCCCTAAAGAGTTTATACC	CGATGCGTCTGAGCACACTCGT

List of primers used for ChIP-qPCR

Target	Forward primers	Reverse primers
BS1-2	ATGCCAATTGCATCATAGCC	TCCTGGAATAGGACCACAGG
BS3-4	AGGCTAAAGCTACGGGGAAA	TGACAAGCAGGAAGAAATGC
BS5-6	GAGGATTGTTCAACCCTGGA	GATGATCCCGTCTTGTCAGAA
BS7	GAGGGGGAGAGGAGAGAGAA	TTTCGCATCACATTTATCAGC

Table S3. Primers used for qPCR, pX330 plasmid construction and ChIP-qPCR

Development • Supplementary information

Supplementary references

Oosterveen, T., Kurdija, S., Alekseenko, Z., Uhde, C. W., Bergsland, M., Sandberg, M., Andersson, E., Dias, J. M., Muhr, J. and Ericson, J. (2012). Mechanistic differences in the transcriptional interpretation of local and long-range Shh morphogen signaling. *Dev Cell* **23**. 1006-1019.

Development • Supplementary information
